# A deformation energy-based model for predicting nucleosome dyads and occupancy

**DOI:** 10.1038/srep24133

**Published:** 2016-04-07

**Authors:** Guoqing Liu, Yongqiang Xing, Hongyu Zhao, Jianying Wang, Yu Shang, Lu Cai

**Affiliations:** 1 The Institute of Bioengineering and Technology, Inner Mongolia University of Science and Technology, Baotou, 014010, China; 2 Computational Systems Biology Lab, Department of Biochemistry and Molecular Biology, Institute of Bioinformatics, University of Georgia, Athens, GA 30602, USA; 3 State Key Laboratory for Utilization of Bayan Obo Multi-Metallic Resources, Inner Mongolia University of Science and Technology, Baotou, 014010, China; 4 College of Computer Science and Technology, Jilin University, Changchun, Jilin 130021, China

## Abstract

Nucleosome plays an essential role in various cellular processes, such as DNA replication, recombination, and transcription. Hence, it is important to decode the mechanism of nucleosome positioning and identify nucleosome positions in the genome. In this paper, we present a model for predicting nucleosome positioning based on DNA deformation, in which both bending and shearing of the nucleosomal DNA are considered. The model successfully predicted the dyad positions of nucleosomes assembled *in vitro* and the *in vitro* map of nucleosomes in *Saccharomyces cerevisiae*. Applying the model to *Caenorhabditis elegans* and *Drosophila melanogaster*, we achieved satisfactory results. Our data also show that shearing energy of nucleosomal DNA outperforms bending energy in nucleosome occupancy prediction and the ability to predict nucleosome dyad positions is attributed to bending energy that is associated with rotational positioning of nucleosomes.

Nucleosome, a fundamental structure unit of chromatins in eukaryotes, consists of a histone octamer and a 147 bp core DNA that is sharply bent and tightly wrapped ~1.7 times around the histone octamer in a left-handed superhelix. The DNA segment between two adjacent nucleosomes is referred to as linker^1^. Nucleosome plays important roles in various cellular processes, such as DNA replication, gene transcription, RNA splicing and recombination, by modulating, in most cases, the accessibility of underlying genomic sequence to proteins^2^,^3^,^4^. For example, depletion of nucleosomes near transcription start sites of genes can assist the binding of transcription factors to their binding sites^5^; Nucleosome organization at replication origins affects replication program^6^,^7^,^8^,^9^; Chromatin remodeling and histone modification are required in meiotic recombination^10^,^11^; RNA Pol II density at exons modulated by nucleosome positioning may influence the recruitment of splicing factors to pre-mRNA and splicing pattern^12^,^13^,^14^. Therefore, the identification of nucleosome positions along genomic sequences and the understanding of the underlying mechanism are substantially important for deciphering the chromatin function.

Various factors affect nucleosome positioning. Nucleosome positioning is a kind of protein-DNA interaction, in which amino acid composition and physicochemical properties of proteins play important roles and thus can be used to predict protein structures and protein-DNA interactions^15^,^16^,^17^,^18^. However, histone octamers involved in nucleosome formation are compositionally conserved and structurally stable, suggesting that major signals contributing to nucleosome positioning are likely to be encoded in DNA sequence. Indeed, the intrinsic preference of DNA sequence was shown to be crucial in nucleosome positioning^19^. The internal signals encoded in DNA sequence include the ~10-bp periodicity of dinucleotides, nucleosome-forming motifs and DNA deformability^19^,^20^,^21^,^22^,^23^,^24^. For example, ~10-bp periodically occurred AA/TT/TA/AT dinucleotides that oscillate in phase with each other and out of phase with ~10-bp periodic CC/GG/CG/GC dinucleotides can facilitate the bending of DNA around histone octamers^19^,^20^,^21^,^22^. Besides, external factors^25^,^26^,^27^ such as chromatin remodelers, DNA methylation, RNA polymerase II binding, etc., were shown to play an important role in nucleosome positioning.

Segal *et al*. proposed that the intrinsic sequence preference can explain ~50% of the *in vivo* nucleosome positions^19^. However, Zhang *et al*.^27^ argued that intrinsic DNA-histone interactions are not the major determinant of nucleosome positioning *in vivo* and the nucleosome pattern inside the genes arises primarily from statistical ordering induced by a RNA polymerase II-associated barrier that regulates transcription initiation, although the nucleosomes immediately flanking the nucleosome free regions at transcription start sites are directed, at least in part, by positioning signals encoded in underlying genomic sequences, such as dinucleotide 10-bp periodicity^19^,^25^.

Mavrich *et al*.^25^ proposed the statistical positioning model, in which the nucleosome-depleted regions at transcription start sites act as barriers from which nucleosomes are positioned like an array, independent of sequence preference or other external factors, toward both directions with a decreasing stability. Furthermore, the distance between the 5′ and 3′ nucleosome free regions (NFRs) was shown to control the strikingly organized nucleosome ordering in intragenic regions in yeast, which is likely to regulate gene expression at the level of transcription elongation^28^,^29^. For example, small genes present a clear periodic packing between the two bordering NFRs while larger genes show a fuzzy nucleosome positioning. Vaillant *et al*.^30^ also demonstrated that a thermodynamical model of nucleosome assembly at equilibrium established on grand canonical description of the nucleosomal positioning can account well for the regular statistical positioning of nucleosomes in genes. Zhang *et al*.^31^ however, argued that nucleosome organization around 5′ ends of genes can be explained by ATP-facilitated statistical positioning rather than intrinsic DNA-histone interactions, statistical positioning and transcription-based mechanism. Regardless of the debate, the significant similarity between the *in vitro* and *in vivo* maps of nucleosome organization^20^ and considerable studies^32^ that predicted nucleosome occupancy with high accuracy based merely on the DNA sequence or its physical properties demonstrated the sequence-dependency of nucleosome positioning.

In recent years, experimental mapping of genome-wide nucleosome organization has been obtained for several model systems^33^,^34^,^35^,^36^,^37^,^38^, such as *Saccharomyces cerevisiae*, *Caenorhabditis elegans*, *Drosophila melanogaster* and *Homo sapiens*, but the mechanism of nucleosome positioning still remains elusive. A variety of models have been proposed for predicting nucleosome occupancy that are classified into categories of bioinformatics^19^,^39^,^40^,^41^,^42^,^43^,^44^,^45^ and energetics of nucleosomal DNA^46^,^47^,^48^,^49^,^50^,^51^,^52^,^53^,^54^. Bioinformatics models learn various sequence features, such as dinucleotide distributions and oligonucleotide motif frequency from a large quantity of nucleosomes^39^,^40^,^41^,^42^,^43^. Among the bioinformatics models, machine learning methods could efficiently discriminate two extremes in nucleosome forming ability, but show poor accuracy in classifying the sequences that have moderate ability to form nucleosomes and are less capable of predicting the centers of nucleosomes. Several bioinformatics models, however, show an increased ability to predict dyad positions of nucleosomes. For example, DNA bendability matrix, which reflects the phase relationships between various dinucleotides within the helical period, was used for predicting nucleosome positions with one base-pair resolution^44^. In another bioinformatics model, the periodic distribution of several most important dinucleotides for nucleosome positioning was used to establish a scoring function without considering the positions of the dinucleotides in nucleosomes and predicted the dyads of nucleosomes reconstituted *in vitro* successfully^45^.

There are a number of energetics models designed to predict nucleosome formation energy, nucleosome occupancy and positions^46^,^47^,^48^,^49^,^50^,^51^,^52^,^53^,^54^. A model^51^ that took into account the deformations of DNA helical twist, roll and tilt achieved a moderate correlation between its prediction and experimental nucleosome occupancy (R = 0.45, P < 0.0001, on yeast chromosome III). Nucleosomal DNA in the model was viewed as an unshearable elastic rod, neglecting the energy cost required for DNA shearing in nucleosome formation. Another model focused on the contribution of roll and slide to the nucleosomal geometry and documented that the bending of nucleosomal DNA is ascribed largely to roll while the shear of nucleosomal DNA largely to slide^52^. This roll-and-slide mechanism was extended and described mathematically by Bishop in an ideal superhelix form^55^. Morozov *et al*.^53^ presented a model in which the DNA geometry is allowed to get relaxed from its initial conformation and hence the total elastic energy include two parts, sequence-dependent DNA elastic potential and non-specific histone-DNA interaction energy designed to penalize deviations of nucleosomal DNA from the ideal superhelix. Most intriguing in the model is the ability to predict nucleosome dyad positions, while less concern was given to discriminate the contribution of DNA bending and shearing to nucleosome formation and positioning. In addition, Deniz *et al*.^56^ presented a model in which physical parameters, such as stiffness and structural parameters, were derived from atomistic molecular dynamic simulations, and found that regions around transcription start sites and termination sites have high flexibility and nulceosome depleted regions are characterized by high deformation energy of DNA.

In this report, we present an improved deformation energy model to predict nucleosome positioning based merely on DNA physical properties, focusing on the contribution of both bending and shearing of DNA to nucleosome formation. The model utilized the overall structure constraints (such as superhelical curvature and pitch) of nucleosomal DNA to calculate elastic energy and achieved a good performance in predicting both nucleosome dyad positions and occupancy.

## Materials and Methods

### Materials

We used the experimental data of normalized-nucleosome occupancy (*in vitro* and *in vivo*) across the genome (sacCer1 version) of *Saccharomyces cerevisiae* from Kaplan *et al*.^20^, *in vitro* nucleosome map of *Saccharomyces cerevisiae* from Zhang *et al*.^27^ (GSE15188), ten DNA sequences used to assemble nucleosomes *in vitro* from Cui *et al*.^57^, nucleosome center positioning (NCP) score/noise ratio from Brogaard *et al*.^34^, and the complete genome sequences of *Saccharomyces cerevisiae* (sacCer1 and sacCer2 version) retrieved from UCSC (http://genome.ucsc.edu/). The top 500 nucleosomal DNA sequences with highest NCP ratios^34^ were retrieved from the yeast genome (sacCer2 version) by using corresponding genomic position information. Genomic positions of 3600 recombination hotspots and transcription start sites (TSS) and transcription end sites (TES) of 5015 validated transcripts in yeast were taken from Pan *et al*.^58^ and Lee *et al*.^33^, respectively. Genomic positions of 47 consensus sequences (ACS) in autonomously replicating sequences (ARS) at replication origins, which can be downloaded from SGD database, were provided in Supplementary information (Table S6). In addition, the *in vivo* nucleosome map^23^ (adjusted nucleosome coverage) and corresponding genome (WS170 version) of *Caenorhabditis elegans* were downloaded from UCSC (http://genome.ucsc.edu/). A dataset of nucleosome-forming and nucleosome-inhibiting sequences in *Saccharomyces cerevisiae* and *Drosophila melanogaster* defined in a previous study^59^ was used to make a two-class prediction. The genome (dm3 version) of *Drosophila melanogaster* was downloaded from UCSC.

### Deformation energy calculation

There are two major kinds of DNA deformations^1^, bending and shearing, which are subjected to two global structure constraints in nucleosome formation, radius (equivalent to overall bending angle) and pitch of superhelix geometry, so we formulated these two deformations separately to explore their respective role in nucleosome positioning prediction.

The geometry of DNA double helix is required for the deformation energy calculation. We adopt the system recommended by Cambridge Convention^60^ to describe the geometry of DNA double helix, in which each base pair is viewed as a rigid board, and its position relevant to its neighbor is specified by six degrees of freedom, such as roll, tilt, twist, slide, shift and rise.

In principle, nucleosomal DNAs should have lower deformation energy than the linkers and, accordingly, the deformation energy of DNA is calculated to predict nucleosome positioning using an elastic model in this study. Numerous studies using molecular dynamics and statistical dynamics supported the elasticity of DNA sequence^48^,^61^,^62^,^63^ and DNA elastic models achieved a great success in modeling protein-DNA interactions^63^,^64^. DNA bending and shear in the formation of nucleosomes were well illustrated in the crystal structures of nucleosome core particles^65^,^66^ and some studies have also mathematically formulated and successfully modeled the deformation of nucleosomal DNA^1^,^55^. Therefore, in this model, DNA is viewed as shearable elastic rod and nucleosomal DNA deformation is viewed as forced bending and shearing. For calculational simplicity , the torque is assumed to be uniformly distributed along the DNA. We consider DNA bending to be analogous to bending a rod of multiple segments with variable stiffness. For a bending force exerted by the histone octamer on a segment of the DNA, the deformation energy at each step along the sequence depends on both the corresponding dinucleotide flexibility and the phasing of the dinucleotide with respect to the dyad.

The ideal DNA superhelix^1^ that best fits the core DNA in the nucleosome core particle (NCP147) has a radius of 41.9 Å and a pitch of 25.9 Å. Curvature in the ideal DNA superhelix derives equally from roll and tilt, whereas, as shown by the crystal structure of NCP147 (1kx5), the curvature of NCP147 DNA stems predominately from roll^1^. The crystal structure indicates that the two relatively straight 9-bp terminal segments of NCP147 contribute little to the curvature of actual superhelix^1^. Thus, only the central 129-bp segment that yields a curvature of 579° for the ideal superhelix is considered in deformation energy calculation. To evaluate the possible effect of two terminal 9-bp ends in a nucleosomal DNA on nucleosome positioning, we did some analysis using a 147-bp window in deformation energy calculation with corresponding curvature of 600°, which was inferred from the ideal superhelix^1^. Unless stated, we used a sliding window of 129 bp in deformation energy calculation.

The deformation energy of a nucleosomal DNA is formulated below. In our model, it is assumed that DNA bending in a nucleosome is derived from roll and tilt, and DNA shear from slide and shift.

At dinucleotide step *i* (integer number),


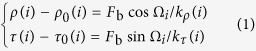


Thus, the bending energy can be evaluated by


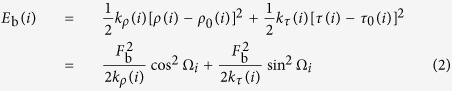


where 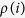 and 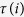 are, respectively, the actual roll and tilt angle at dinucleotide step *i*, and 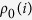 and 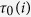, which are dependent on the dinucleotide at step *i*, are, respectively, the roll and tilt without torque; 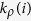 and 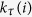 are the dinucleotide-dependent force constants; Ω_*i*_ is the cumulative helical twist at the center of step *i*, counted from the dyad point. For 147-bp nucleosomal core DNA, its structure is symmetrical with respect to the dyad that is located at the central nucleotide, and the dinucleotide steps from the dyad are labeled as 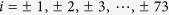 towards downstream and upstream directions. The step ±1 are half step away from the dyad, thus the cumulative helical twist is calculated as follows:


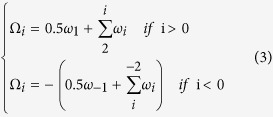


The bending energy for the central *L*-bp segment of a nucleosomal DNA is the sum of corresponding dinucleotide steps:


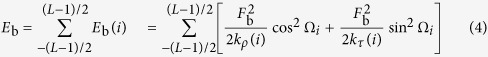


where *L*, a positive odd number, is less than or equal to 147.

In the Equation (4), *F*_b_ is determined by its relationship with the bending angle of the core DNA. The central 129-bp part of the nucleosomal core DNA bends around histone octamer about 579° (*α*) under the stress of *F*_b_, and the *α* is the total contribution of roll and tilt for each step. We therefore have


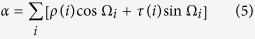


Combining Eq. (1) and Eq. (5) leads to


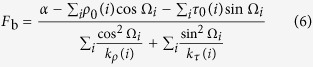


The ability of a nucleosomal DNA to form nucleosome is generally anti-correlated with the torque imposed on the nucleosomal DNA. The relationship of the torque with base-pair-step angles and phase of the step relative to the dyad is readily seen from the Equation (6): If the signs of 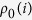 and cos Ω_*i*_ are the same, their contribution to the torque is negative; otherwise, their contribution is positive. Similar is hold for the tilt. Appropriate phasing of dinucleotides with respect to dyad axis can increase the contribution of roll and tilt angles of dinucleotide steps to the total bending angle of the core DNA, thereby reducing *F*_b_ that is inverse-correlated with the nucleosome-forming ability. For example, for a DNA tract, the dinucleotides with high positive rolls occurred at the positions with high cos Ω_*i*_ and the dinucleotides with low negative rolls occurred at the positions with low cosΩ_*i*_ would facilitate its nucleosome formation.

Nucleosomal DNA shear is caused by slide and shift. We use the following formulas to describe the relationship between shearing force *F*_s_ and deviations of the two degrees of freedom from their respective equilibrium state,


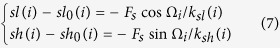


The ideal superhelix of nucleosomal DNA has a radius of 41.9 Å and a pitch of 25.9 Å. The 25.9 Å pitch results from slide and shift, in which the former contributes to most of the pitch. For the central 129-bp part of nucleosomal DNA, we thus have


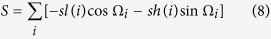


where *S* is the displacement of superhelical DNA along the screw axis. By analyzing the ideal superhelical path of nucleosomal DNA, we have *S* = 41.96 Å.

Combining equations (7) and (8) leads to


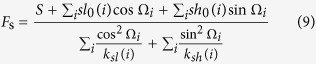


where *k*_*s*_(*i*) is the force constant, *s*(*i*) and *s*_0_(*i*) are the slides of the step *i* with and without stress of *F*_s_ respectively. Similar with the formulation of bending energy, the deformation energy that corresponds to the shearing of nucleosomal DNA is


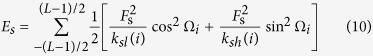


The total deformation energy is estimated by


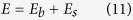


Average deformation energies per base-pair step for a sequence segment of 129 bp with respect to bending energy, shearing energy and total energy are computed throughout this study.

In our deformation energy model, DNA-histone interactions along nucleosome DNA is not considered, and this is not likely to have severe influence on our results, as the previous study showed that the sequence of NCP147 particle after free relaxation with constrained ends strikingly resembles the true NCP147 path^64^.

The empirical parameters of our model for deformation energy calculation consist of force constants (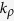, 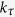, 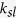
and 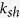) and equilibrium structural parameters (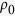, 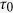, 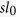, 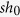 and 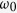) for 10 dinucleotides (complementary dinucleotides are considered to be the same). The above dinucleotide-dependent parameters were estimated by using the protein–DNA crystal structures in the latest NDB database (http://ndbserver.rutgers.edu/, update of Aug.1, 2014) and listed in Table S1. We extracted all the B-DNA structures from protein–DNA complexes, and excluded the base-pairs with chemical modification considering that it may influence the base-pair step structure. The DNA structures were described by the aforementioned six degrees of freedom, which were obtained by using 3DNA program^67^. Dramatically distorted base-pair steps that at least one of its geometric parameters deviate more than 2 Standard Deviation from its mean were excluded from our dataset to avoid possible non-harmonic effect. The equilibrium structural parameters were the average values of the local geometric parameters for each dinucleotide type. The force constants were computed by inverting the covariance matrix of deviations of local geometric parameters from their average values^53^,^68^. Nevertheless, two modifications made in the calculation that differ from others’ should be noted. First, in the covariance matrix calculation, the base-steps were counted once even for self-complementary dinucleotides that might be counted twice in others’ studies^53^,^68^. If the step parameters are counted twice, the covariance that involves tilt and shift would be mis-estimated. For example, both variances (an element of covariance matrix) of tilt and shift is over-estimated to ~four times original variances while those of other parameters remain unchanged if they are counted twice given that tilt and shift change their signs when the direction of z axis is changed (Fig. S1). This would further lead to the loss of comparable meaning between the different force constants. Second, equilibrium tilts were calculated separately for complementary dinucleotides because the tendency of tilt angle to open toward the dinucleotide in one strand differs from its tendency to open toward the complementary dinucleotide. As shown by statistical results (Table S1), equilibrium tilts for complementary dinucleotides differ considerably, and this may influence calculation results because the sign of equilibrium tilts combined with the twisting phase is important for bending force needed to bend it around histone octamer (see Equation 6). Note that different equilibrium tilts for complementary dinucleotides can result in bending energy difference if calculated separately on Watson and Crick strand. In order to obtain a consistent result between Watson and Crick strand, we made a modification to the dinucleotide equilibrium parameters including tilt and shift (see the illustration below Table S1). The modified equilibrium tilts (or shift) for complementary dinucleotides differ only in sign, resulting in the same bending energy (or shearing energy) between Watson strand and Crick one.

Besides, equilibrium tilts and shifts for the self-complementary dinucleotides (AT, TA, CG, GC) were assigned zero in light of following considerations. For tilt, its expectation is zero because it has equal ability to open toward either of two self-complementary dinucleotides. For shift, although there is possible anisotropy in its ability to open toward either of two self-complementary dinucleotides, its average from a sufficient sampling is about zero as the probability that shift is considered to be positive or negative (counted on complementary strand) is the same. These considerations differ from previous works in which self-complementary dinucleotides in sequences were counted twice and the averages for tilt and shift equal zero since the signs of tilt and shift are changed when dinucleotides are counted in an opposite direction on the complementary strand.

In the present model, we represented base-pair step twist at each step in the DNA by modified sequence-dependent equilibrium twists 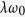, in which 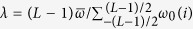, 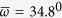 is the average step twist for the 1kx5 X-ray crystal structure of nucleosome-bound DNA, and *L* is length of DNA segment for which deformation energy is calculated. The modification to the twist reflects its sequence-dependent alteration along the sequence under the constraint that the twist sum along *L*-length DNA segment is 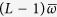, which approximately equals that of crystal structure of the nucleosome.

### Nucleosome occupancy estimates

The probability of a nucleosome dyad being at any site along underlying DNA and nucleosome occupancy were predicted by using a grand canonical model^53^,^69^ (Supplementary Information). Nucleosomes are viewed as a many body system and described as a grand canonical ensemble, in which bulk histone octamers may adsorb on or desorb from DNA. The dynamical assembly of histone octamers along DNA is controlled by a thermal bath, chemical potential of histone reservoir, steric hindrance between adjacent nucleosomes and the non-homogeneous adsorbing potential (eg. deformation energy of DNA). Steric exclusion (nucleosome overlap is not allowed) is considered in the model and deformation energy is used as input. We focus on DNA-directed nucleosome positioning mechanisms, and some other factors that play important roles in nucleosome organization *in vivo*, such as DNA binding molecules and remodelers, are not considered in this study. The partition function in the model is calculated using a dynamic programming method^53^,^69^.

## Results

### Prediction of nucleosome dyad positions

Compared to flanking linker sequences, a nucleosomal DNA should have a lower deformation energy. To test our model, we calculated deformation energy profiles for 10 nucleosomal DNA sequences, for which precise positions of 20 nucleosomes assembled *in vitro* along the DNA sequences are known (See Supplementary Information for primary DNA sequences). As shown in Fig. S2, the calculated local deformation energy minima coincide well with the nucleosome dyad positions. We also show that local bending energy minima coincide well with the nucleosome positions (including 5S DNA) with average uncertainty less than 2 bp (Fig. S3). Contrary to DNA bending, shearing energy of the nucleosomal DNA cannot successfully indicate nucleosome dyad positions (Fig. S4). The spikes of the dyad probability of a nucleosome (probability of a nucleosome to center at a position) calculated on bending energy also show good overlap with the experimental dyad positions of the nucleosomes (Fig. S5), indicating bending energy is a good indicator of nucleosome dyad positions.

**Figure 1:**
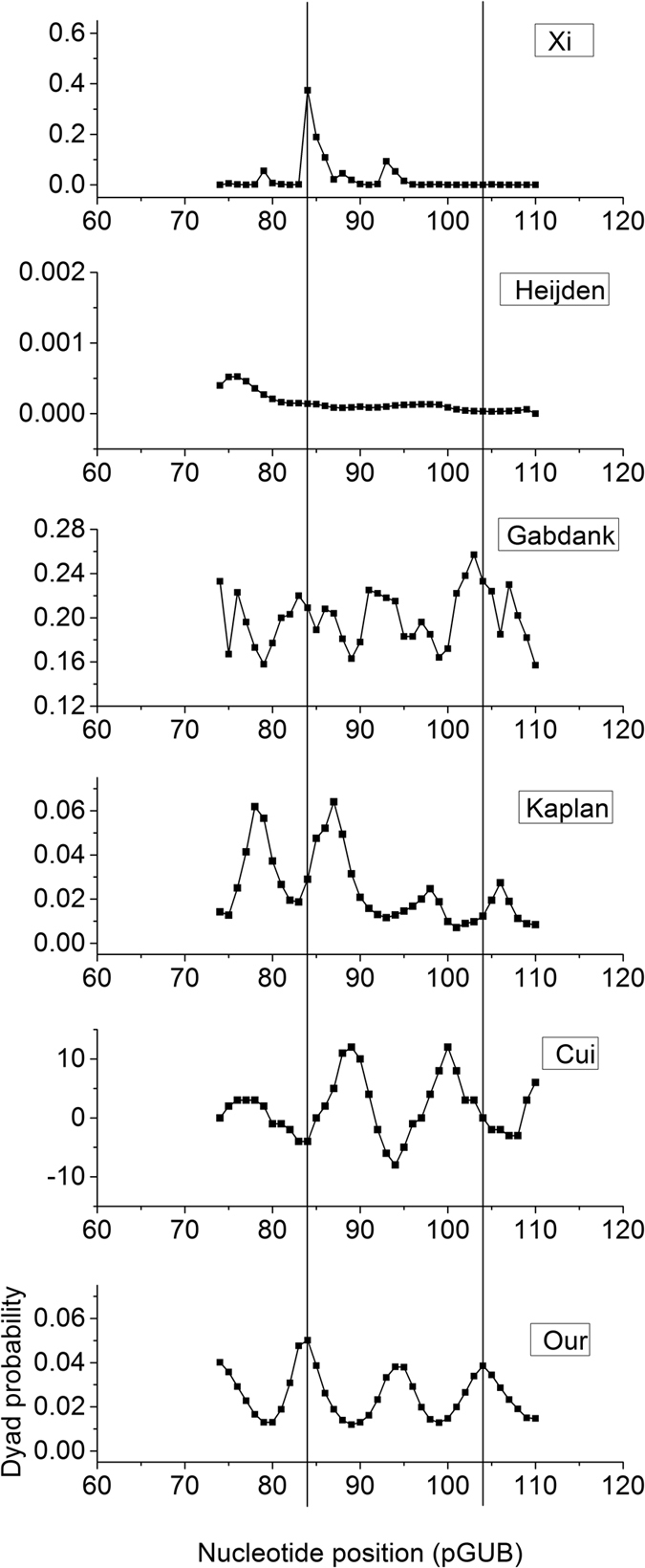
Calculated dyad probability for a nucleosomal DNA sequence (pGUB). Vertical lines denote experimentally-determined nucleosome dyad positions. Predictions with published models are provided for comparison. Parameters used in the models: Our model (*τ* = 0.35, *β* = 35), Kaplan’s model (*τ* = 0.1, *β* = 1), Heijden’s model (B = 0.2, *p* = 10.1 bp, N = 146 bp). The results for other nucleosomal DNA sequences were provided in supplementary information (Fig. S4).

We compared our results with some published models that can suggest nucleosome dyad positions (Fig. S5). Of the analyzed 20 nucleosome positions, 19 were successfully predicted with less than 2 bp uncertainty by our local maxima of calculated dyad probability. Cui *et al*.’s model^57^ successfully predicted 16 dyad positions, Kaplan *et al*.’s model^20^ 16, Gabdank *et al*.’s model^44^ 10, Heijden *et al*.’ model^45^ 5 and Xi *et al*.’s model^70^ 4 with less than 2 bp uncertainty. The only one unsuccessful prediction of our model is for a nucleosome positioned on 5S oocyte sequence (dyad position at135 bp). For this nucleosome, Cui *et al*.’s model also made a poor prediction, while Kaplan *et al*.’s and Gabdank *et al*.’s models suggested a possible dyad near the experimental position. For another nucleosome positioned at 159 bp on 5S oocyte, however, Kaplan *et al*.’s and Gabdank *et al*.’s models made poor predictions while Cui *et al*.’s and our models predicted it well. Another remarkable difference of our prediction with others is that our model accurately predicted two nucleosomes on pGUB sequence while Cui *et al*.’s and Kaplan *et al*.’s models made poor predictions (Fig. 1).

In Fig. S3, the calculated bending energy oscillated with a periodicity of 10–11 bases. The adjacent deformation energy minima with 10 bp intervals between them indicate possible translational positioning of a nucleosome. The 10-bp periodicity of dinucleotides encoded in nucleosomal sequences makes the DNA adopt a single rotational setting on the histone surface and this restricts a nucleosome to translational settings separated by 10 bp, which keep the direction of DNA bending (rotational positioning)^71^. Consistent with this, nucleosome reconstitution experiments demonstrated that the alternative positions from the dyad with increments of 10 bp are physically eligible because they differ mildly in the stability of the complexes^24^,^72^.

Brogaard *et al*.^34^ produced a unique map of 67,543 nucleosome positions with base pair resolution in yeast, allowing two neighboring nucleosomes to overlap by no more than 40 base pairs. To test the ability of our model to predict nucleosome center, we also obtained an average deformation energy profile for the genomic regions centered at the top 500 nucleosomes with highest nucleosome center positioning (NCP) score/noise ratio. Although there is no consistent energy profile for the nucleosomal DNA segments, it is obvious that deformation energy minimum tends to occur at the centers of the nucleosomes (Fig. S6). Besides, deformation energy profile exhibits ~10-bp periodicity oscillation and gradient descent of amplitude from the center towards each side, as the amplitude indicates the strength of rotational positioning, which becomes weaker with linkers that have no rotational positioning signal entering the energy-calculation window of 129 bp. Results of power spectrum analysis conducted with Fast Fourier Transform algorithm show that the ~10-bp periodicity in energy profile is significantly stronger in nucleosome core regions (central 129 bp) than in flanking regions (flanking 19 bp at each end) (Fig. S7).

### Nucleosome occupancy prediction

We tested the performance of our model by predicting nucleosome occupancy along yeast chromosome III based merely on DNA bending energy or shearing energy. Although shearing contributes only ~30% to overall deformation energy, the shearing energy shows a much better performance in nucleosome occupancy prediction than bending energy (Table 1). In light of this, we predicted nucleosome occupancy using shearing energy in the rest of this study. Our data also indicated that 147-bp window-based deformation energy calculation yielded a very similar energy profile with the 129-bp window-based results in terms of shearing energy (Pearson correlation: R = 0.934, P < 0.0001) and bending energy (R = 0.936, P < 0.0001). However, the inclusion of two relatively straight 9-bp terminal segments of a nucleosome in the model has some negative impact on the bending energy-based prediction (147-bp window-based results in Table 1), suggesting algorithms used to simulate the DNA bending in a nucleosome may benefit from the exclusion of the two straight ends. In contrast, the inclusion of the two ends has no effect on the shearing energy-based nucleosome occupancy prediction (Table 1).

The profiles of shearing energy are relatively flat (low variance) and the profiles of total deformation energy and bending energy are similar since the main contribution to both the deformation energy and its variance is given by the bending. Accordingly, shearing energy is not significant for the identification of nucleosome dyad positions. However, the shearing energy improves the performance of predicting nucleosome occupancy, because the shearing energy is able to capture the relative strength of nucleosome forming ability along a DNA sequence. This will be discussed later in detail.

The genome-wide deformation energy in yeast is calculated by using a sliding window of 129 bp with a step of 1 bp along the genome and then genome-wide nucleosome occupancy is estimated. As shown in Table 2, the predicted nucleosome occupancy has higher correlations with *in vitro* occupancy than with *in vivo* occupancy as expected since *in vitro* nucleosome occupancy is affected only by sequence properties and possible steric hindrance. The ability of our model in genome-wide prediction of nucleosome occupancy is improved considerably than the preliminary one that was based only on bending deformation^54^. Although less successful than Kaplan *et al*.’s model (R = 0.84) which is a scoring function method based on training data, our model generated a higher genome-scale correlation (R≈0.80) between prediction and *in vitro* experimental nucleosome occupancy than many other energetics models, such as Miele *et al*.’s model^51^ (R = 0.45 for chrIII) and Locke *et al*.’s model^69^ (R = 0.75). Our model also outperforms a bioinformatics model called NuPoP^70^ (Table 2). It is worth noting that there is an unexpected extremely poor prediction for the tenth chromosome (chrX) of the yeast genome (Table 2). After a careful check on the original data of Kaplan *et al*.^20^, we found the correlation (R = 0.227) between their prediction and the *in vitro* nucleosome occupancy for chrX is also unusually low, and the genome-scale Pearson correlation (R = 0.84) recalculated on the data differs from the one (R = 0.89) reported in their paper^20^. It is theoretically possible that their data for chrX available on the internet is likely to be an artifact of possible errors made during experiments or merely in uploading the data. However, possible reasons for the low correlation need to be investigated in future because we also observed similar results using the dataset of Zhang *et al*.^27^ (Table 2).

One question needs to be discussed here is how does the grand canonical model response to the nucleosome coverage change. The nucleosome coverage in the chromatin fibers reconstituted *in vitro*^20^ is about 30%, which is much lower than that *in vivo*. It seems that, in the modeling, the chemical potential in the grand canonical model needs to be adjusted to capture the nucleosome coverage difference. However, the best predictions were obtained using the same parameter values (*τ* = 0.001, *β* = 15) in the modeling of both *in vivo* and *in vitro* data of Kaplan *et al*.^20^ (Table 2). It is probably because the difference between *in vitro* and *in vivo* data is dominated by non-sequence factors, such as remodelers and RNA polymerase binding *in vivo*, rather than by average nucleosome coverage. By this we mean that the non-sequence external effects *in vivo* override nucleosome coverage difference. When applied to another *in vitro* map^27^, in which 1:1 histone-to-DNA mass ratio was employed, differing from 0.4:1 ratio used in Kaplan *et al*.^20^, our model obtained the best prediction with a different chemical potential-related parameter (*τ* = 0.01, *β* = 15, Table 2). This suggests that modeling of different *in vitro* maps that differ in nucleosome concentration is dependent on the chemical potential in our model. The prediction of the data of Kaplan *et al*. is more successful than that of Zhang *et al*. (Table 2), suggesting the lower histone-to-DNA mass ratio used in Kaplan *et al*.^20^ may enable intrinsic DNA preference to play a more important role in nucleosome positioning. Note that Locke *et al*.’s modeling^69^ of the data of Zhang *et al*.^27^ is slightly better than ours, which probably results, at least in part, from the normalizing algorithm applied to the sequence reads by Locke *et al*.^69^.

Our model is based largely on physical properties of DNA sequences, and thus its applicability to other genomes can be expected. To test this, we applied the model to *Caenorhabditis elegans*, and achieved a moderate correlation between our prediction and the experimental nucleosome occupancy *in vivo* (Table 3), which is comparable to Kaplan *et al*.’s result (R = 0.47 for chrII) and higher than the result obtained by NuPoP model^70^ (Table 3). The prediction of nucleosome occupancy in *Caenorhabditis elegans* (Table 3) is less accurate than in yeast (Table 2), which is likely to be caused by some non-sequence factors, such as chromatin remodelers, RNA pol II binding, etc., which are known to be much more complex in *Caenorhabditis elegans* than in yeast. The prediction of the nucleosome organization on the mitochondria genome of *Caenorhabditis elegans* is better than that of other chromosomes (Table 3), suggesting nucleosome positioning on the mitochondria genome may depend more strongly on sequence preference than other chromosomes.

**Figure 2:**
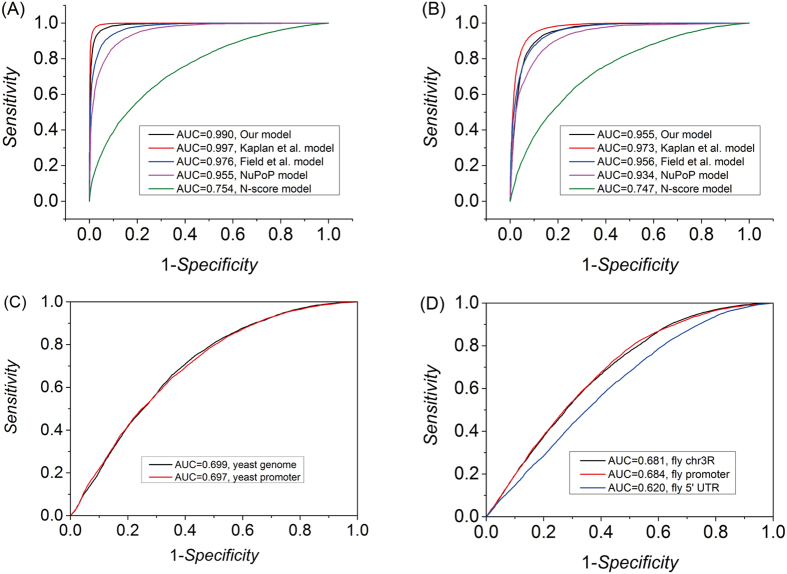
The performances of models in classifying nucleosome-forming and nucleosome-inhibiting sequences were evaluated by ROC curves. (**A**) test on yeast nucleosome-enriched and nucleosome-depleted regions defined on Kaplan *et al*.’s *in vitro* map; (**B**) test on yeast nucleosome-enriched and nucleosome-depleted regions defined on Kaplan *et al*.’s *in vivo* map; (**C**) test on yeast nucleosome-forming and nucleosome-inhibiting sequences taken from Liu *et al*.^59^, which were defined on Lee *et al*.’s *in vivo* map^33^; (**D**) test on fruit fly nucleosome-forming and nucleosome-inhibiting sequences taken from Liu *et al*.^59^, which were defined on Mavrich *et al*.’s *in vivo* map^36^.

We also compared our model with published models by classifying nucleosome-forming sequences and nucleosome-inhibiting sequences (see Supplementary Methods for classification procedure). The results show that our model performs very well in discriminating nucleosome-enriched regions from nucleosome-depleted regions in yeast (Fig. 2A,B). Boltzmann model-based prediction (Supplementary Methods) achieved the same classification performance (AUC = 0.99 for *in vitro* data and AUC = 0.95 for *in vivo* data) as Grand canonical model-based prediction (Fig. 2A,B), suggesting that the high performance of Grand canonical model is not because its parameters were fitted to the *in vitro* map. We also carried out a classification using *in vivo* nucleosome data of yeast and fruit fly in the same way as described in a previous study^59^ (see Supplementary Information for method details). As compared with the prediction results of eight models^59^, our model has a moderate performance (Fig. 2C,D), ranking about fourth among the models. NuPoP^70^ performs slightly worse than our model when predicting nucleosome data of Kaplan *et al*.^20^ (Fig. 2A,B), but it outperforms our model when predicting nucleosome data of Lee *et al*.^33^ (Fig. 2C in this study and Fig. 4 in Liu *et al*.^59^), suggesting nucleosome dynamics and external factors *in vivo* can influence prediction accuracy. For *Drosophila melanogaster*, our prediction accuracy is comparable to NuPoP (Fig. 2D). Some models may have a highly variable ability in the prediction of nucleosomes in different genomic regions^59^. Our model, however, displayed a relatively stable prediction performance for genome-wide regions, promoters and 5′ UTRs , suggesting our model is not biased towards a particular type of genomic regions. Taken together, although in some cases our model performs worse than some previously published models, its application to different species, such as yeast, nematode and fruit fly, achieved a moderate or even much better performance.

Our model, as many other models did, also successfully reproduced the depletion of nucleosomes around transcription start sites and transcription end sites of verified transcripts (Fig. S8). Specifically, nucleosomes are distributed more scarcely at upstream promoter regions of highly-transcribed genes than that of lowly-transcribed genes, which is consistent with the finding that nucleosome occupancy at promoters is inversely correlated with transcriptional activity^19^,^33^,^35^. Nucleosome depletion at the downstream of transcription end sites is stronger for highly-transcribed genes than lowly-transcribed genes.

We focus on physical properties of DNA sequences that influence nucleosome positioning in this study, and therefore no attempt was made to simulate the long-range ordering of nucleosome arrays around TSS and TES *in vivo*, which is largely determined by strong energy barriers along genomic sequences and nucleosome concentration. The absence of statistical positioning of nucleosomes *in vitro* experiment can be explained by the lack of strong energy barriers (such as transcription factor and RNA polymerase II binding to TSS regions) and low nucleosome concentration. As shown by previous models^28^,^29^, even if DNA sequence effect is neglected, a grand canonical model with artificially imposed strong energy barriers at gene ends could successfully simulate the regularly positioned nucleosomes in genes^29^.

In addition to gene transcription, some other fundamental molecular processes, such as DNA replication and recombination, are also subjected to local chromatin structure. DNA replication starts from replication origins. At replication origins, a short essential consensus sequence (ACS) characterized by low nucleosome occupancy provides the binding site for the origin recognition complex^73^,^74^. Lack of nucleosomes at recombination hotspots might be a prerequisite for double-strand-break (DSB) formation, which initiates meiotic recombination^58^. As discovered before^6^,^58^,^73^,^74^, we also observed nucleosome depletions at the replication origins and recombination hotspots based on our sequence-dependent prediction (Fig. S9), suggesting the nucleosome depletion at replication origins and recombination hotspots is likely to be determined largely by DNA physical properties.

## Discussion

### Some remarks on force constants of dinucleotides

In our model, the sequence-dependent force constants for dinucleotides have a crucial effect on the prediction of nucleosome occupancy, as different sets of force constants (Table S1–S3) show diverse performance in the prediction (Table S4). The poor prediction based on Olson *et al*.’s parameters^68^ (force constants and equilibrium structural parameters) is likely to arise from the force constants in that the substitution of our force constants for theirs achieved a high prediction success (Table S4). A simple correlation analysis also shows that our force constants have diverse correlations with others and the poorest correlation is with Olson *et al*.’s force constants for tilt (Table S5).

Since force constant is so crucial in deformation energy calculation, there is a great demand for correct estimation of force constants, especially determination of the relative magnitudes of the force constants for different dinucleotides taking various effects listed below into account. Firstly, deformation of inter-dinucleotides in tetramers is affected by its adjacent nucleotides^75^,^76^. For example, tilt and roll seem to be affected weakly by the flanking nucleotides, while all the other parameters are sensitive for at least some dinucleotide steps^76^. Furthermore, the influence of the flanking nucleotides on individual dinucleotide steps is variable. The observation^76^ that flanking nucleotides can also influence the variances of the parameters further demonstrated the presence of impact of flanking nucleotides on the estimation of force constants that are inversely correlated with sequence flexibility. Because dinucleotide steps are influenced by flanking nucleotides, the increasing number of experimentally determined structures of DNA-protein complexes can help to improve the estimation of oligonucleotide parameters as well as DNA deformation energy calculation. Secondly, some tetramers exhibit multiple conformational substates, resulting in multimodal or non-Gaussian distributions of structural parameters^76^. This is hence likely to affect the estimation of force constants, particularly the equilibrium values of the parameters. Thirdly, the flexibility of a DNA segment is also dependent on its length^61^,^77^, and this may play an important role in the dynamics of relatively small-sized DNA molecules that are frequently used as targets *in vitro* experiments of nucleosome reconstitution. In addition, different kinds of methods may help to estimate force constants more accurately. For example, force constants can be inferred from the contour surfaces of slide and shift^75^, or molecular dynamics simulation^56^,^78^,^79^.

### Factors that determine nucleosome dyad positions

It has been reported that in salt dialysis reconstitution of nucleosomes the (H3∕H4)_2_ tetramer first occupied the central 74-part of the nucleosomal DNA and then the H2A/H2B dimers bind with the tetramer to wrap the remaining 73 bp into a complete nucleosome^80^,^81^. A bioinformatics model based on periodic distribution function over a window of 74 bp predicted the 601 dyad successfully^45^, implying that periodicity in the central 74-bp part might define the nucleosome dyad position in reconstitution reactions. Our 75-bp window-based calculation, however, does not give any further improvement in prediction of dyad positions.

Our data imply that shear deformation along the superhelix axis of nucleosomal DNA in the form of slide and shift plays a better role than bending deformation in determining the coarse-grained nucleosome occupancy, while the bending deformation in the form of roll and tilt defines well the fine-scale dyad positions of nucleosomes within a coarse-grained nucleosome positioning region. Periodical occurrence of dinucleotides with large absolute values of static roll and tilt in phase-dependent positions in a nucleosome determines rotational positioning of the nucleosome. In other words, rotational positioning-associated properties are important in prediction of the dyad position of a nucleosome.

The potential of a narrow-range region (>147 bp) of a DNA sequence to form nucleosome can be predicted successfully by shearing energy and where to place the dyad of a nucleosome is determined, at least largely, by bending energy. Here we don’t mean bending energy of a DNA segment has no link to its nucleosome formation potential, as our bending energy-based prediction of nucleosome occupancy also has a good correlation (Table 1), though not as strong as shearing energy, with genome-wide experimental occupancy. Shearing energy is unable to indicate the dyad positions of nucleosomes because rotational positioning of a nucleosome, which is closely related to the dyad of the nucleosome due to phasing effect, is deterimined largely by properties of roll and tilt rather than slide and shift, and the phase-dependent periodicity of slide and shift that contribute to the shearing of nucleosomal DNA is relatively weak as reflected by crystal structures of nucleosomes^1^.

**Figure 3:**
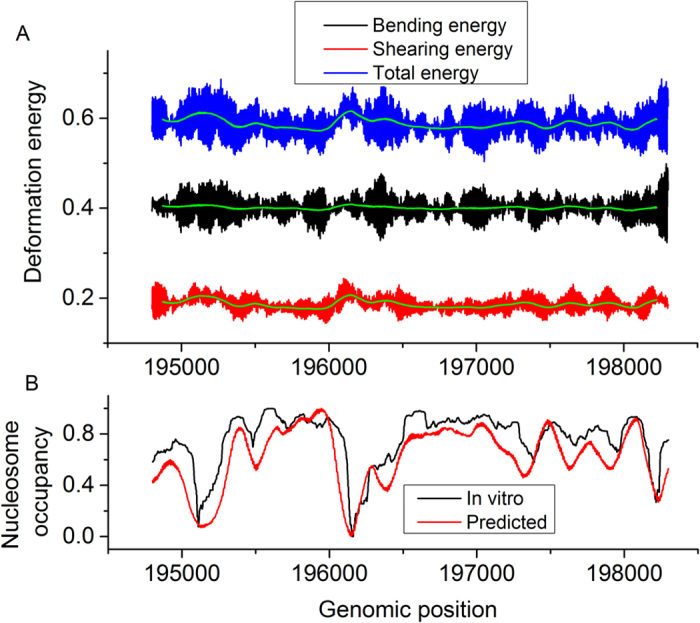
Illustration of the roles of bending energy and shearing energy in nucleosome occupancy estimation. The local mean (green line) of shearing energies over a sliding window of 147 bp has a higher variance along DNA sequences than that of bending energies (5.8E-05 vs 8.5E-06), leading to the higher performance of shearing energy in nucleosome occupancy prediction than bending energy. Note that local mean of deformation energy profile is anti-correlated with nucleosome occupancy. The predicted nucleosome occupancy was based on shearing energy. Both predicted and *in vitro* nucleosome occupancy were normalized to the range of 0–1 for comparison.

What is the reason for our result that shearing energy can predict much better the nucleosome occupancy than bending energy and genome-scale nucleosome occupancy is predicted by shearing energy slightly better than total deformation energy? These results seem difficult to understand because bending energy that accounts for a major part of total energy has a larger variation along DNA sequences than shearing energy and, in principle, the ability of a DNA region to form nucleosome should be correlated with the total deformation energy better than either bending energy or shearing energy. The biological meaning in the deformation energy profile and methodology can give a answer to this puzzle. First, the amplitude of the bending energy profile is a mark of the strength of rotational positioning, but not a strong indicator of nucleosome occupancy. In the methodology, nucleosome occupancy is estimated by the sum of starting probabilities of a nucleosome over a window of 147 bp (Supplementary Information). This would certainly generate a smoothing effect over energy profile, in which all the energy values including energy maxima and minima are averaged. In other words, nucleosome occupancy is estimated by mean value of deformation energies over a 147-bp window, and the variation of local mean energy, instead of variation of deformation energy per se, measures the nucleosome occupancy alteration along sequences. However, the positions with local energy maxima merely suggest a low starting probability of nucleosome at the positions, but not mean that the positions have low potential to be covered by a nucleosome. If a DNA segment has a strong ability to form a nucleosome and tends to center at a particular position, positions near the possible dyad also have a high probability to be occupied by a nucleosome. Therefore, we propose that it is not wise to use the average value of bending energy profile to estimate the nucleosome-forming ability of sequences, though it indeed performed with a moderate accuracy as shown in Table 2. Most importantly, as shown in Fig. 3, although bending energy variance that is positively correlated with the amplitude of bending energy profile is much higher than that of shearing energy (0.00054 vs 0.00026), the mean of bending energies over a sliding window of 147 bp has less variation along DNA sequences than the mean of shearing energies (8.5E-06 vs 5.8E-05). As a result, total deformation energy-based estimate of nucleosome occupancy is largely determined by shearing energy.

To support our aforementioned explanation, we also predicted nucleosome occupancy with a simple Boltzmann model (Supplementary Methods) from which averaging effect (see Equation 7 in Supplementary Methods) of deformation energy can be clearly seen. We calculated nucleosome occupancy based on bending energy, shearing energy and total energy, respectively, and obtained consistent results with grand canonical model (Table 1), demonstrating the role of local mean of shearing energies in nucleosome occupancy prediction. Our results suggest local mean of shearing energy can capture nucleosome occupancy better than bending energy. Our data also shows that although steric exclusion between nucleosomes is not considered in the Boltzmann model, the prediction result is as good as that of grand canonical model, in which steric exclusion is considered (Table 1, Table 2). This implicates steric hindrance is not as important as previously thought in prediction of nucleosome occupancy. It is not surprising because, contrary to nucleosome organization along single chromosome where steric exclusion must play an important role, nucleosome occupancy in a genomic region is determined by reads numbers of high-throughput experiments such as ChIP-chip and ChIP-seq, which are collected from a pool of cells and thus represent average capacity of DNA to form nucleosomes.

It should be noted that the discrepancy among nucleosome maps obtained in different studies might be caused by several kinds of noise, such as different experimental conditions, sequencing data processing, cell cycle phase, gene transcription rate, nucleosome dynamics and sample variations produced by differences in the growth media^78^. Flores *et al*.^78^ proposed that nucleosomes in most case moves along the one-dimensional DNA fiber and are mainly positioned at specific places in response to strong nucleosome depletion signals, such as intrinsic properties of DNA, the competition of histones with DNA-binding proteins and chromatin-remodelers. The noise in nucleosome occupancy and positioning induced by heterogeneity in MNase-Seq experiments may result in the low accuracy in nucleosome positioning predictive models, particularly when machine-learning methods are used to predict nucleosome data produced under a condition different from training data. Although physical models are not dependent on training datasets, their prediction accuracy can still be influenced by the noise and uncertainties in the experimental data, as shown by the discrepancy of prediction accuracy between the two nucleosome maps^20^,^27^ used in this study. It is therefore possible that the robust set of nucleosome profiles^78^ produced under the same condition may facilitate the comparison of different predictive models. However, as compared with *in vivo* maps, *in vitro* maps are not affected by some noise present *in vivo*, such as cell cycle stage, and can be a proper substrate to investigate sequence dependency of nucleosome positioning.

Although our sequence-dependent model predicted nucleosome occupancy successfully, it does not necessarily mean sequence controls nucleosome positioning. In fact, the relationship between genomic sequences and chromatin structure is bidirectional, leading to the difficulty in distinguishing the cause and effect. Specifically, on one hand, intrinsic DNA sequence properties, as indicated in many studies^19^,^20^, could explain a number of the nucleosome positions in the genome; on the other hand, chromatin architecture could also affect the evolution of genomic sequences^82^,^83^,^84^. For example, sequences are likely to evolve under the selective constraint of nucleosome positioning in eukaryotic genomes, such as the conservation of 10-bp periodicity of dinucleotides in nucleosomal sequences, G + C content conservation in both low and high occupancy regions^84^ and reduced substitution rates in linker regions to maintain correct positioning of nucleosomes via linker-based exclusion signals^82^,^83^.

### URLs

For our data including predicted deformation energy, dyad probability (or starting probability) and nucleosome occupancy and *in vitro* nucleosomal DNA sequences used in this study^57^, see https://github.com/yu-shang/supplementary_data.

## Conclusions

To conclude, we presented a deformation energy-based model for predicting nucleosome positioning using the intrinsic physical parameters of DNA. In the model, both bending energy and shearing energy of nuclesomal DNA were considered, and the parameters in the model, such as force constants and equilibrium structural parameters, were calculated using a large sample of DNA structural data with some modifications. The model successfully predicted nucleosome occupancy along the genome of *Saccharomyces cerevisiae* and dyad positions of *in vitro* assembled nucleosomes. Applying the model to *Caenorhabditis elegans* and *Drosophila melanogaster*, we also achieved satisfactory results. More importantly, our data show that shearing energy of nucleosomal DNA outperforms bending energy in nucleosome occupancy prediction while the ability to predict nucleosome dyad positions is attributed to bending energy that is associated with rotational positioning of nucleosomes.

## Additional Information

**How to cite this article**: Liu, G. *et al*. A deformation energy-based model for predicting nucleosome dyads and occupancy. *Sci. Rep*. **6**, 24133; doi: 10.1038/srep24133 (2016).

## Supplementary Material

Supplementary Information

## Figures and Tables

**Table 1:** Pearson correlation of predicted nucleosome occupancy with experimentally-determined *in vitro* nucleosome occupancy^20^ along the yeast chrIII.

	Grand canonical model	Boltzmann model
Bending	0.634 (0.554)	0.641
Shearing	0.813 (0.809)	0.818
Total	0.795 (0.791)	0.791

Note: all the correlations are significant at the level *P* < 0.0001. The correlation coefficients in the parentheses were based on a 147-bp window used in deformation energy calculation, and other data in this study are all based on a 129-bp window used in deformation energy calculation. ‘Bending’, ‘Shearing’ and ‘Total’ denote the predictions based on bending energy, shearing energy and total deformation energy, respectively. The parameters used in the grand canonical model: bending energy (*τ* = 0.001, *β* = 0.1), shearing energy (*τ* = 0.001, *β* = 15), total energy (*τ* = 0.001, *β* = 1).

**Table 2:** Pearson correlation of predicted nucleosome occupancy with *in vitro* and *in vivo* nucleosome maps of yeast.

Yeast	Grand canonical model		Boltzmann model		NuPoP^70^		Grand canonical model	Boltzmann model
	*in vitro*^a^	*in vivo*^a^	*in vitro*^a^	*in vivo*^a^	*in vitro*^a^	*in vivo*^a^	*in vitro*^b^	*in vitro*^b^
chrI	0.798	0.636	0.806	0.644	0.657	0.577	0.506	0.456
chrII	0.756	0.612	0.751	0.620	0.564	0.490	0.572	0.568
chrIII	0.813	0.653	0.818	0.656	0.652	0.588	0.575	0.527
chrIV	0.793	0.654	0.803	0.672	0.649	0.575	0.572	0.562
chrV	0.812	0.657	0.811	0.665	0.643	0.579	0.598	0.567
chrVI	0.794	0.664	0.801	0.669	0.652	0.586	0.584	0.546
chrVII	0.824	0.686	0.810	0.673	0.647	0.591	0.608	0.574
chrVIII	0.802	0.672	0.811	0.671	0.636	0.578	0.587	0.531
chrIX	0.786	0.651	0.798	0.651	0.642	0.590	0.556	0.514
chrX	0.464	0.428	0.248	0.206	0.197	0.150	0.355	0.168
chrXI	0.796	0.654	0.801	0.662	0.638	0.562	0.626	0.567
chrXII	0.797	0.670	0.805	0.669	0.637	0.576	0.584	0.567
chrXIII	0.810	0.672	0.809	0.666	0.647	0.584	0.617	0.574
chrXIV	0.790	0.634	0.810	0.667	0.635	0.568	0.546	0.527
chrXV	0.802	0.694	0.809	0.669	0.637	0.574	0.591	0.579
chrXVI	0.773	0.649	0.805	0.671	0.641	0.569	0.586	0.577

Note: all the correlations in the table are significant at the level *P* < 0.0001. Nucleosome occupancy in both models was predicted based on shearing energy. The parameters used in the grand canonical model: *τ* = 0.001, *β* = 15 for fitting Kaplan *et al*.’s data, *τ* = 0.01, *β* = 15 for fitting Zhang *et al*.’s data.

^a^Correlations with Kaplan *et al*.’s data^20^.

^b^Correlations with Zhang *et al*.’s data^27^.

**Table 3:** Pearson correlation of predicted nucleosome occupancy with *in vivo* nucleosome map^37^ of *Caenorhabditis elegans*.

	Grand canonical model	Boltzmann model	NuPoP^70^
chrI	0.462	0.470	0.350
chrII	0.474	0.483	0.352
chrIII	0.473	0.479	0.358
chrIV	0.426	0.436	0.335
chrV	0.433	0.445	0.331
chrX	0.468	0.490	0.384
chrM	0.776	0.722	0.547

Note: all the correlations in the table are significant at the level *P* < 0.0001. Nucleosome occupancy in our models was predicted based on shearing energy. The parameters used in the grand canonical model: *τ* = 0.001, *β* = 15.
