# Supplementary material for: A deformation energy-based model for predicting nucleosome dyads and occupancy
Source: Sci Rep. 2016 Apr 7;6:24133. doi: 10.1038/srep24133 (PMC4823781; doi:10.1038/srep24133)
Supplement: Supplementary Information [file srep24133-s1.doc]

**Supplementary Information for**

“A deformation energy-based model for predicting nucleosome dyads and occupancy”

Guoqing Liu^1,3,*^, Yongqiang Xing^1^, Hongyu Zhao^1^, Jianying Wang^1,2^ , Yu Shang^3,4^, Lu Cai^1,*^

**
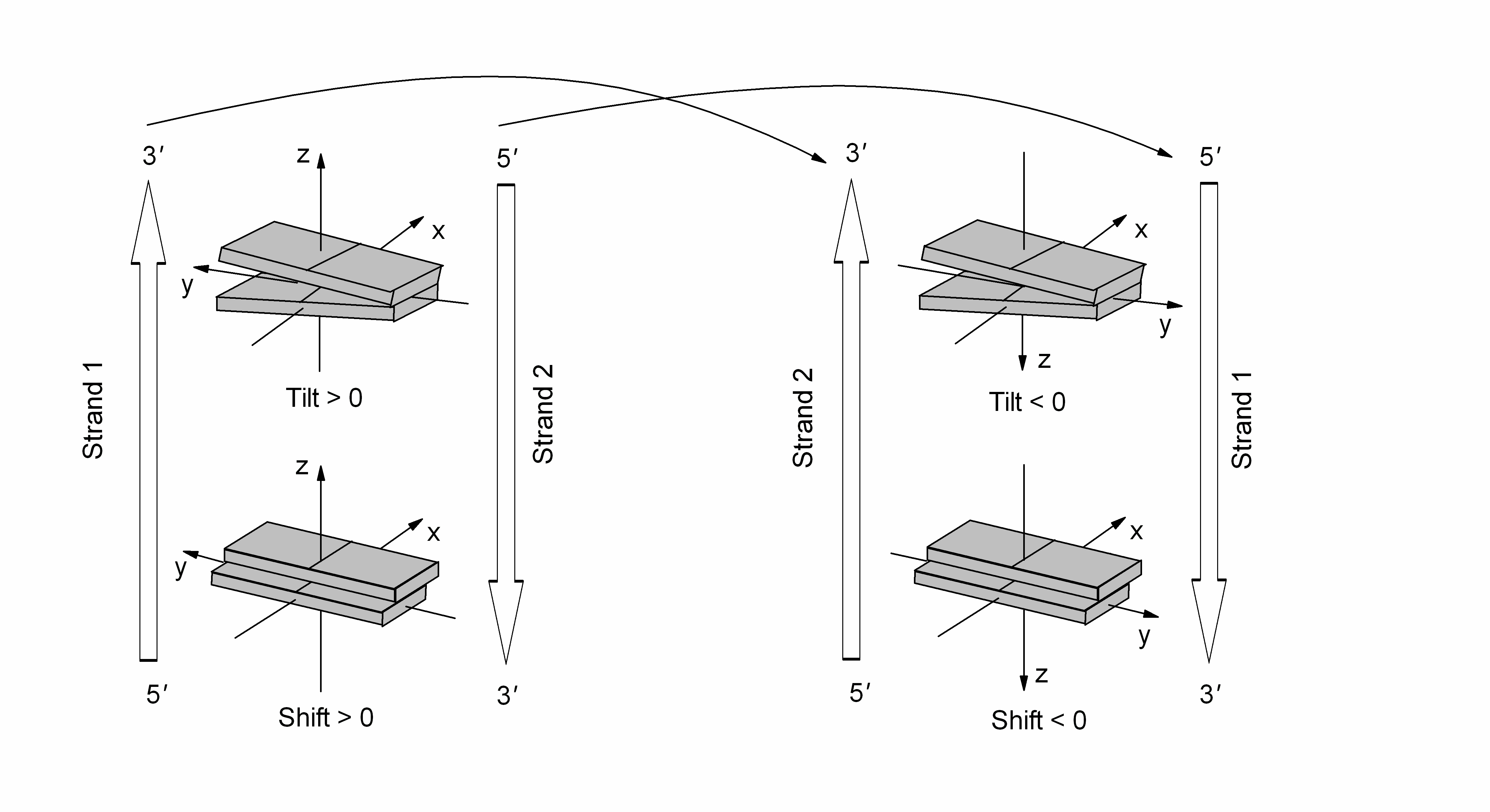
**

Fig S1. According to the Cambridge convention^60^, both tilt and shift parameters change their signs if dinucleotides are counted from the complementary strand (indicated as strand 1).


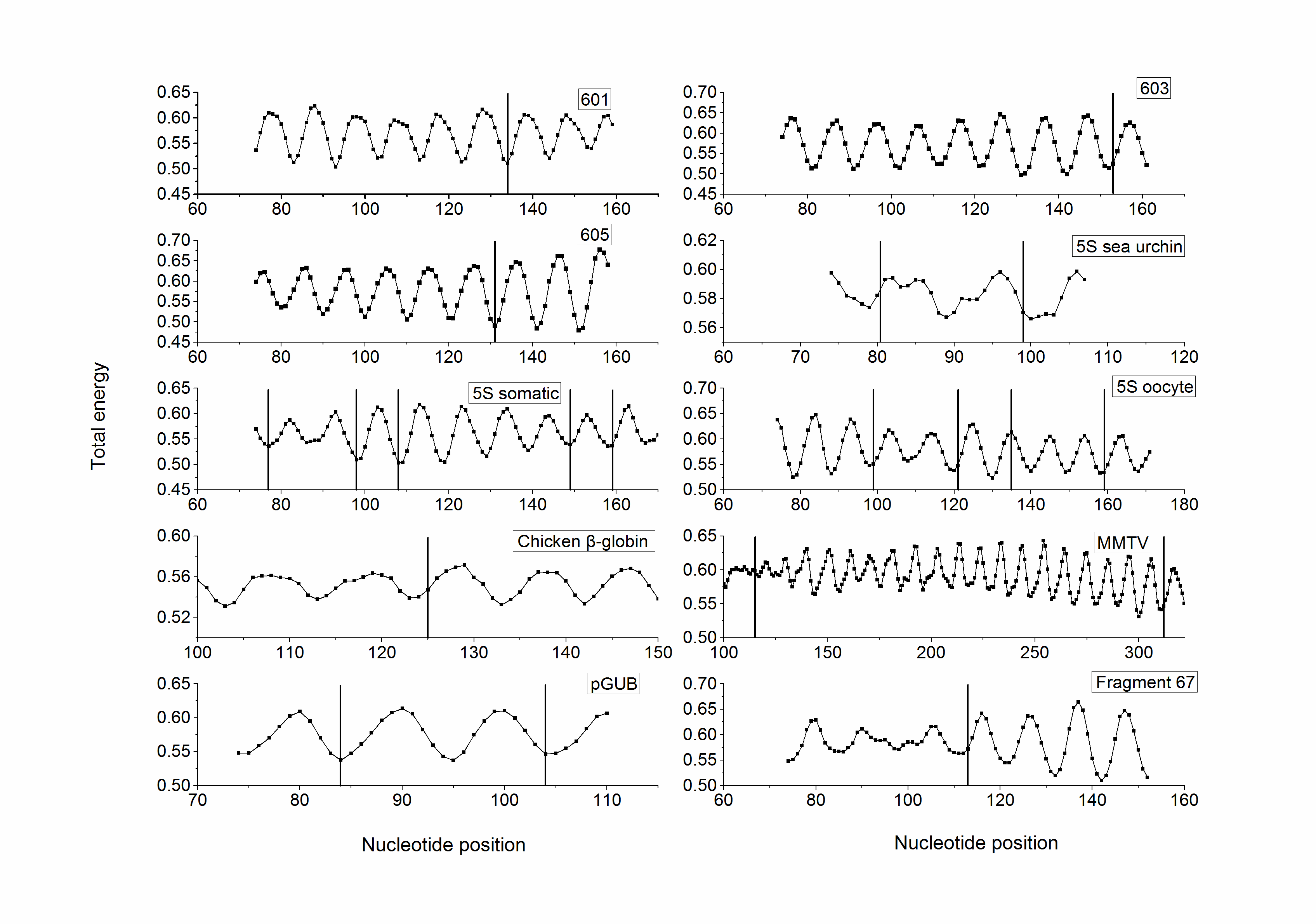
Fig S2. Calculated total deformation energy profile for nucleosomal DNA sequences. Deformation energy corresponds to the 147-bp region centered at the nucleotide position in the abscissa. The first nucleotides in the sequences are denoted as nucleotide position 1. Vertical lines denote experimentally-determined nucleosome dyad positions.


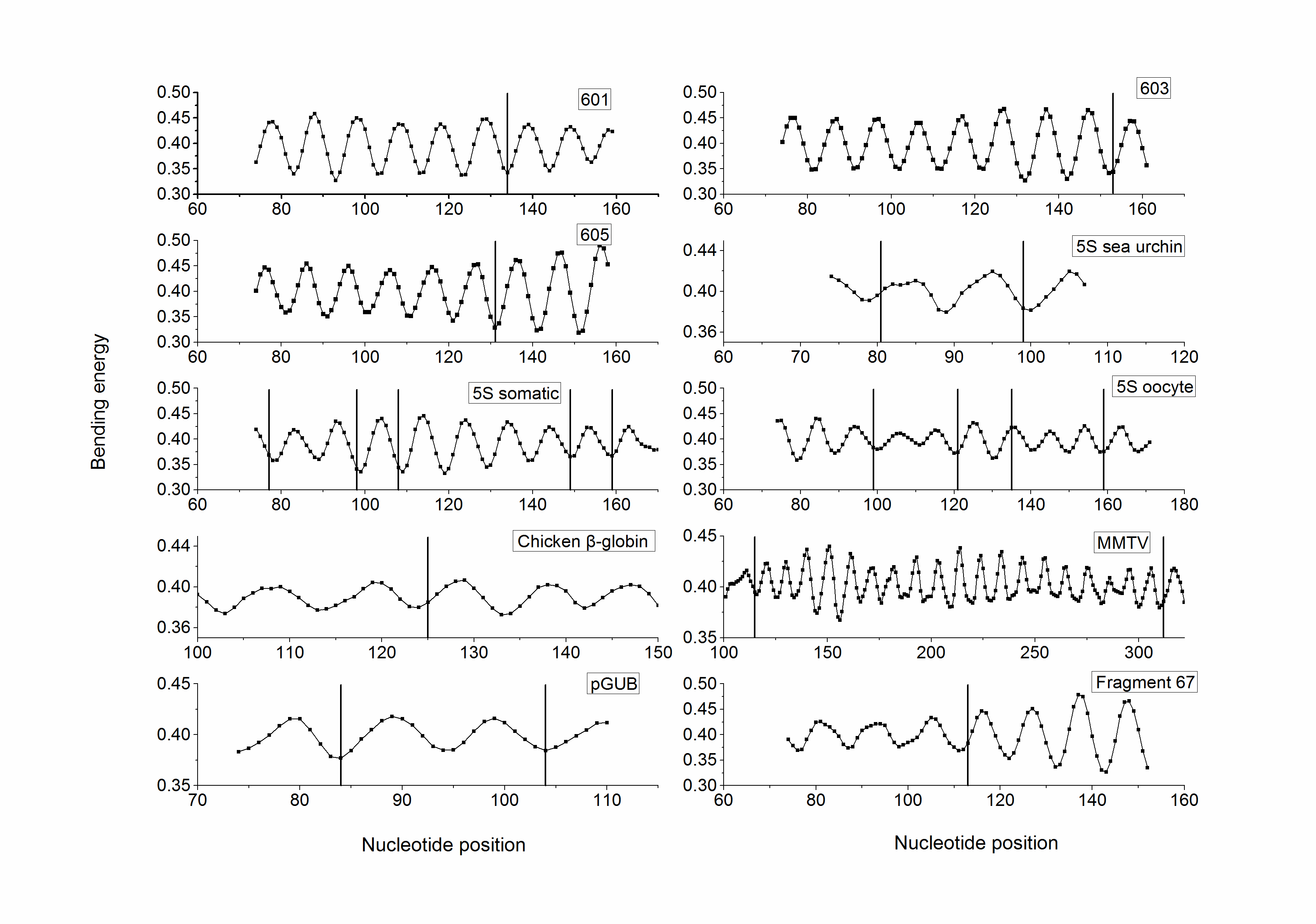
Fig S3. Calculated bending energy profile for nucleosomal DNA sequences. Deformation energy corresponds to the 147-bp region centered at the nucleotide position in the abscissa. The first nucleotides in the sequences are denoted as nucleotide position 1. Vertical lines denote experimentally-determined nucleosome dyad positions.

**
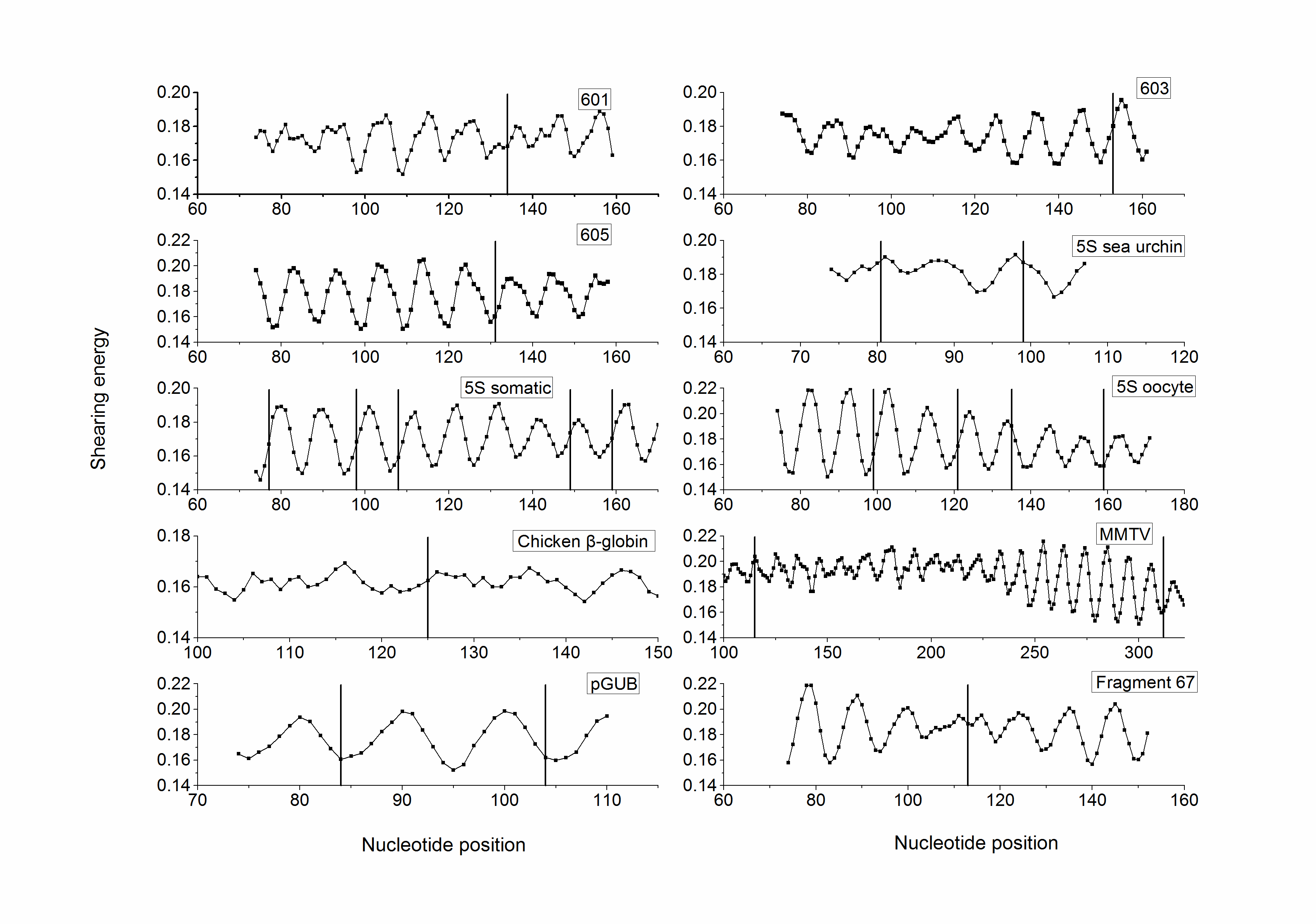
**

Fig S4. Calculated shearing energy profile for nucleosomal DNA sequences. Deformation energy corresponds to the 147-bp region centered at the nucleotide position in the abscissa. The first nucleotides in the sequences are denoted as nucleotide position 1. Vertical lines denote experimentally-determined nucleosome dyad positions.

**
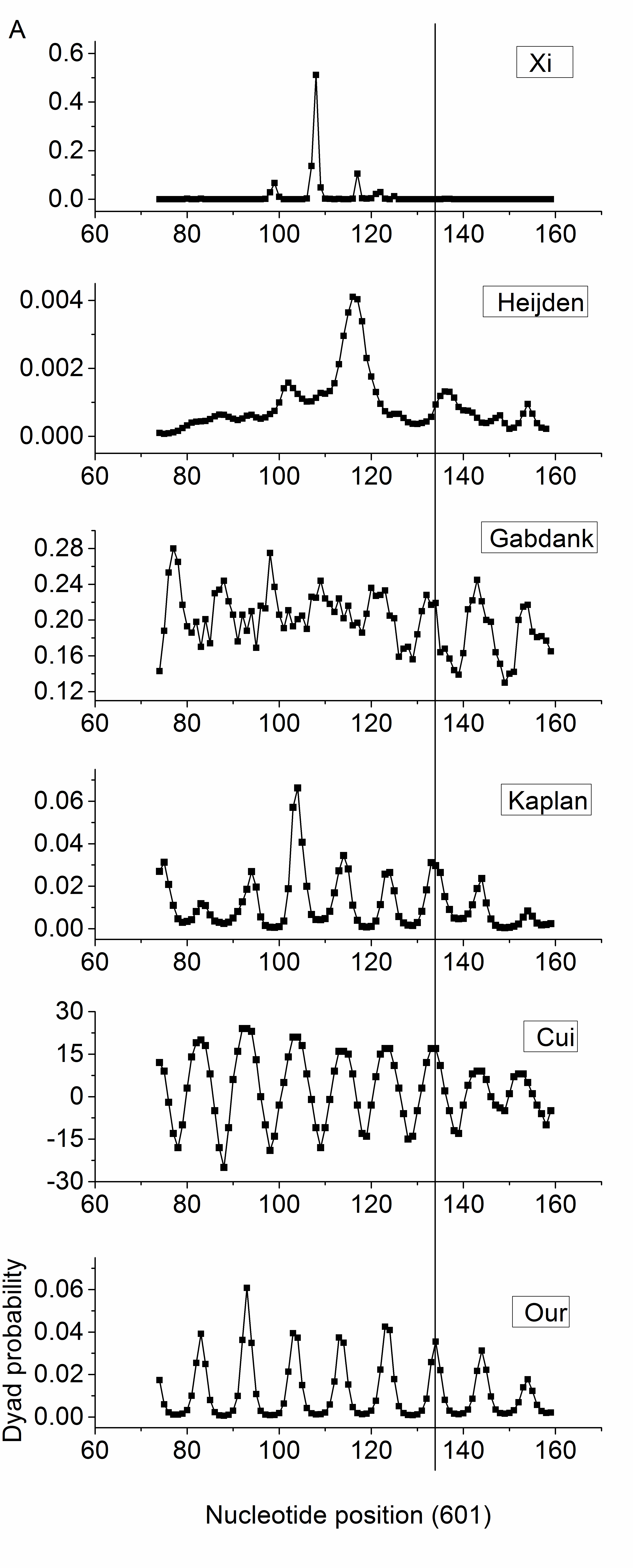

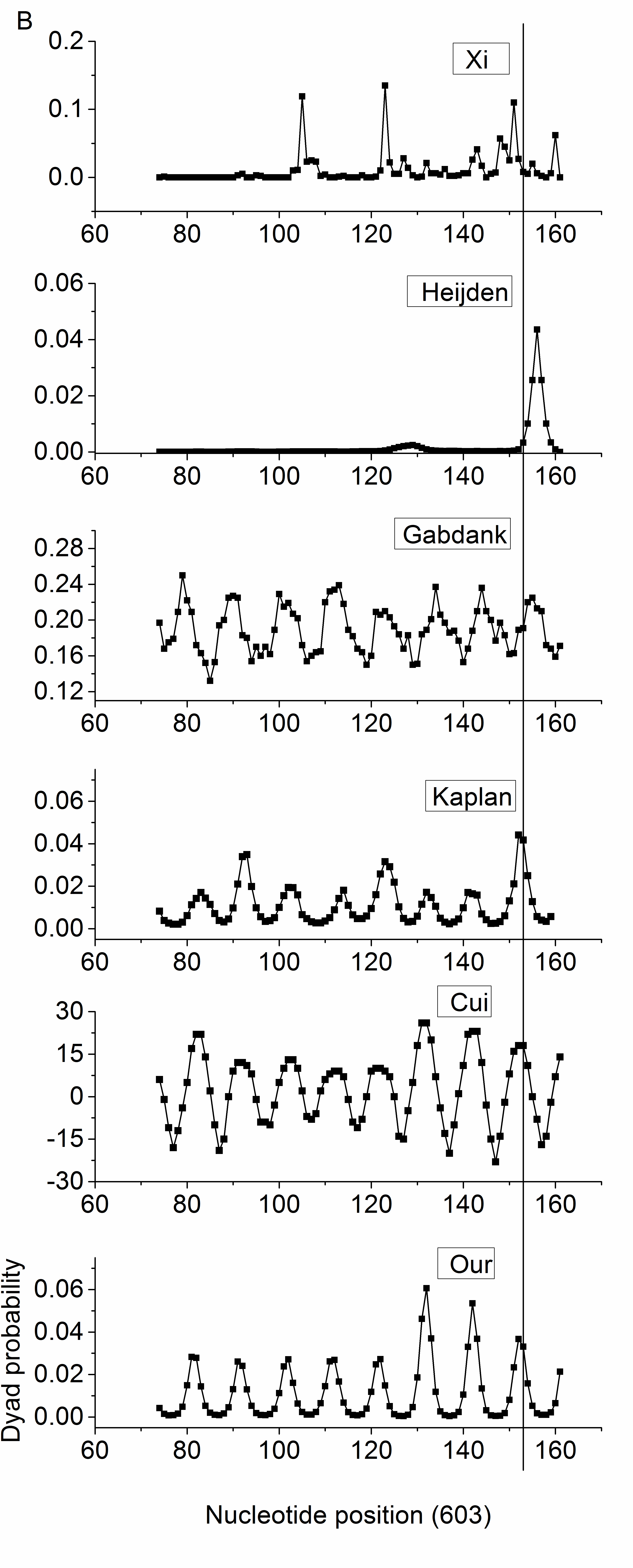
**

**
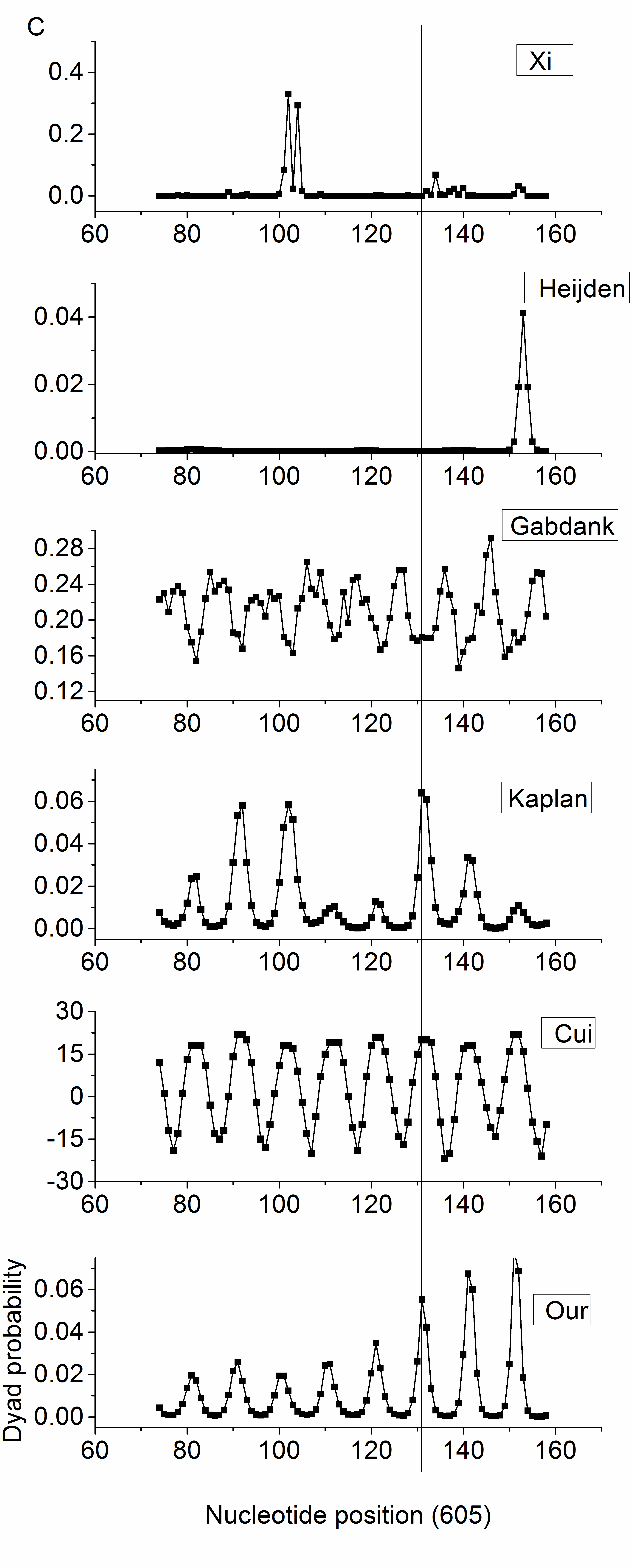

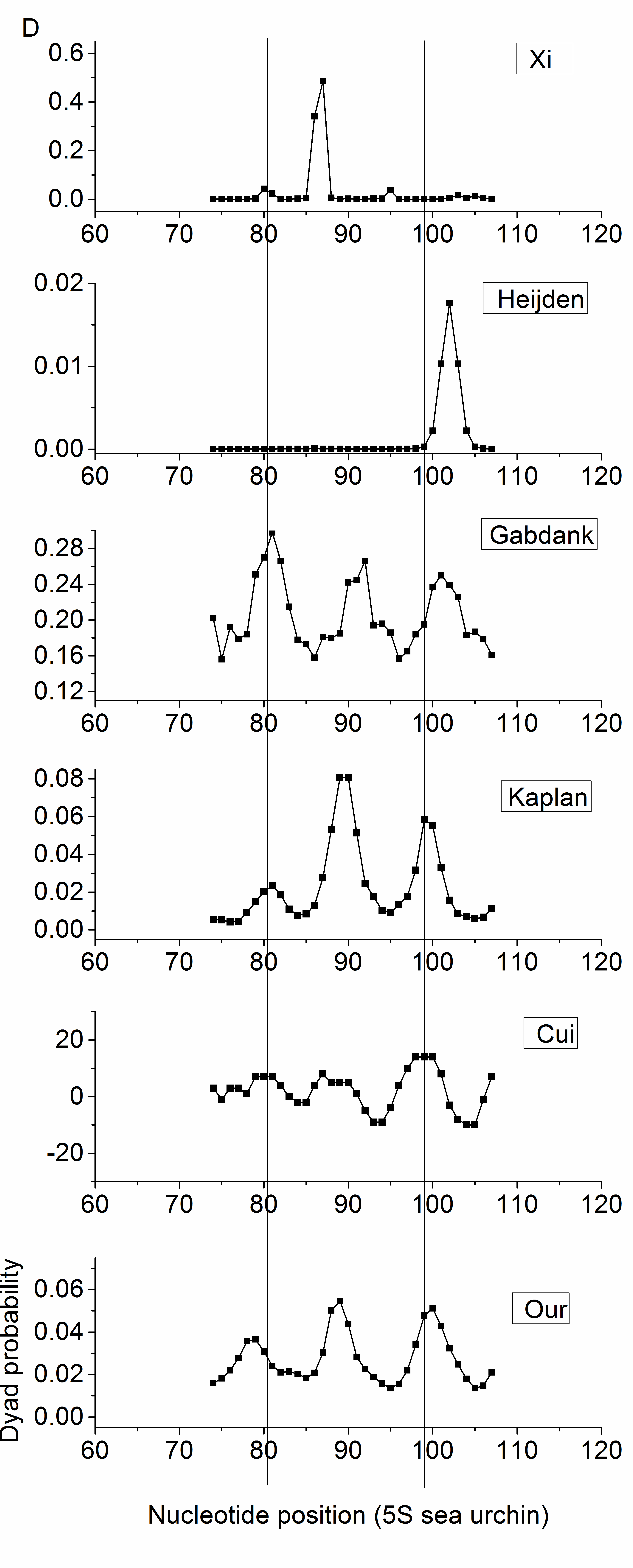
**

**
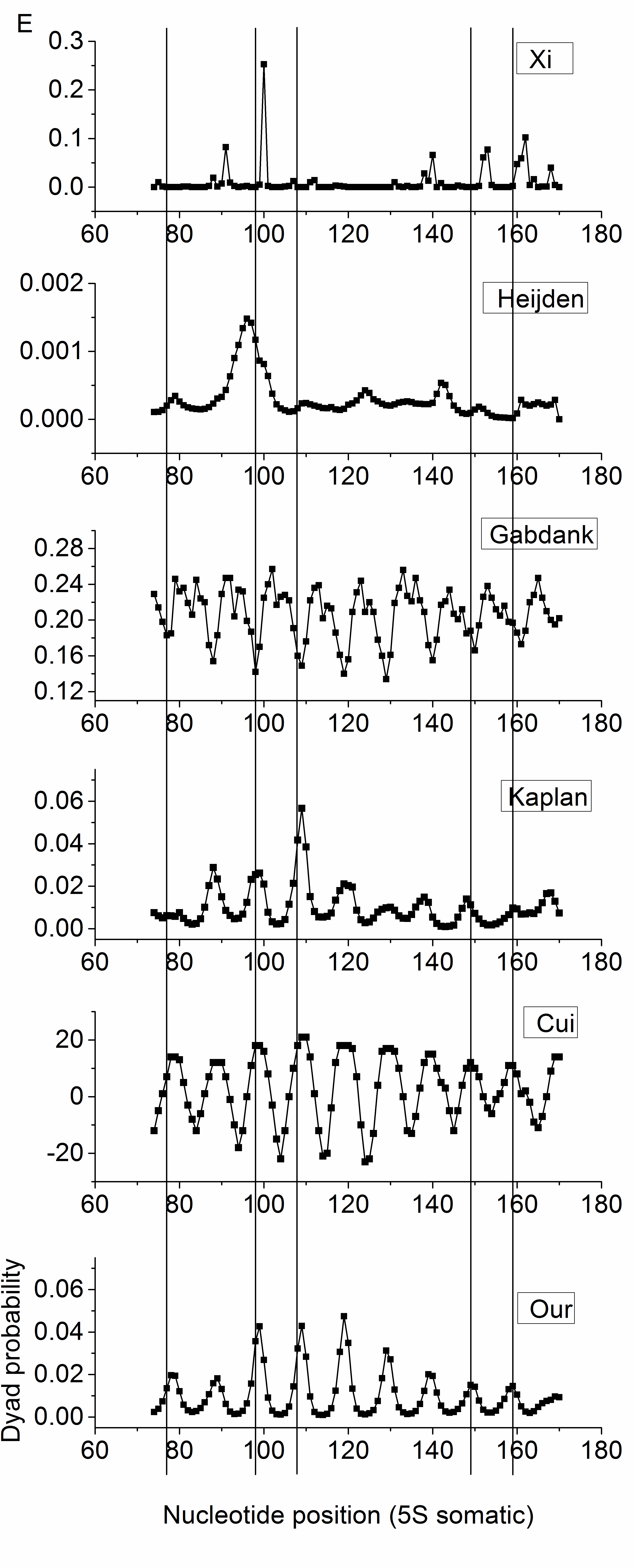

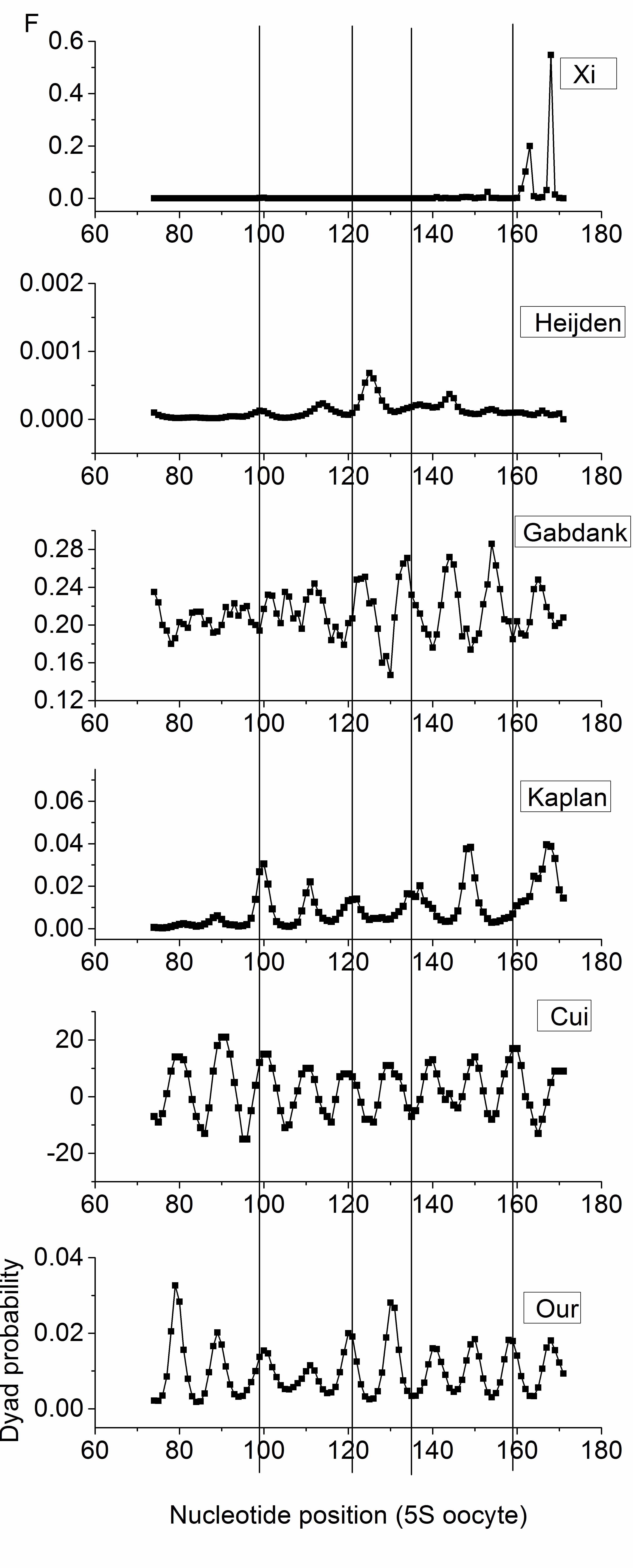
**

**
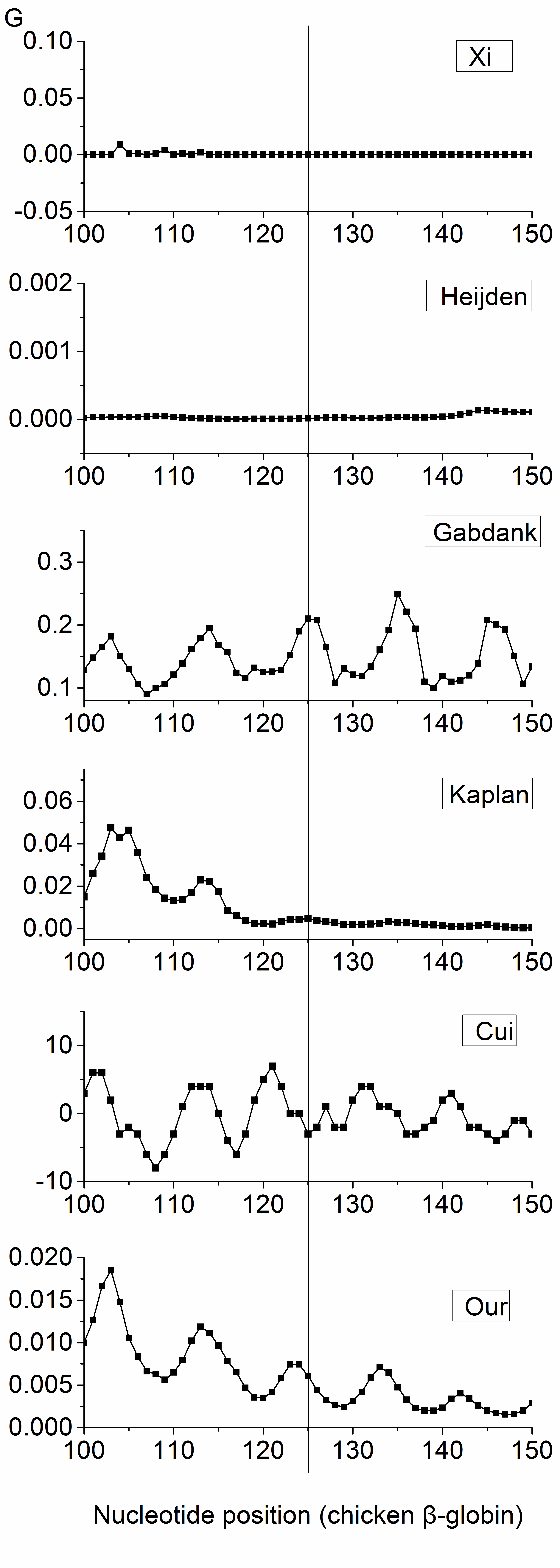

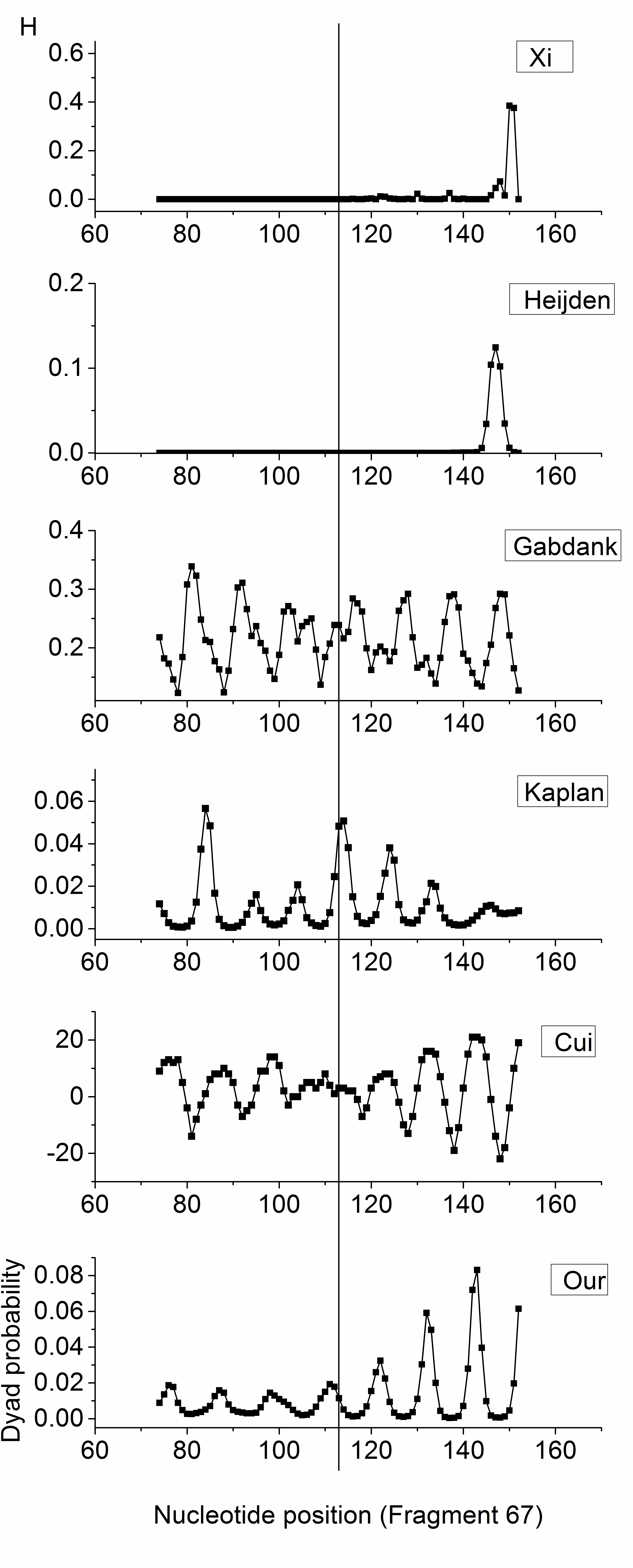
**

**
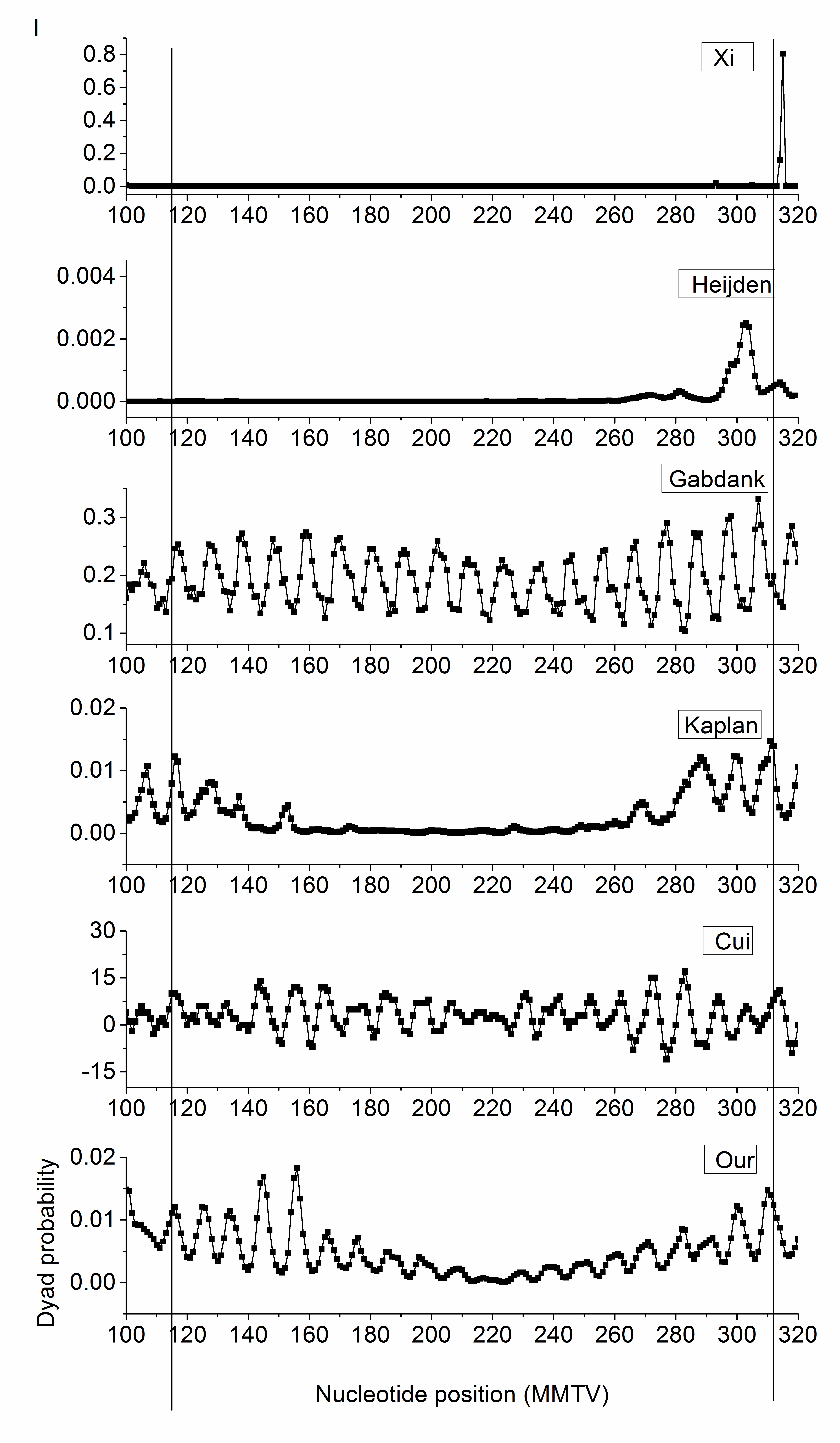
**

Fig S5. Calculated dyad probability for nucleosomal DNA sequences. Vertical lines denote experimentally-determined nucleosome dyad positions. Predictions with published models are provided for comparison. Parameters used in the models: Our model ($\tau=0.35$,$\beta=35$), Kaplan et al.'s model^20^ ($\tau=0.1$,$\beta=1$), Heijden et al.'s model^45^ (B = 0.2, *p* = 10.1 bp, N = 146 bp).

**
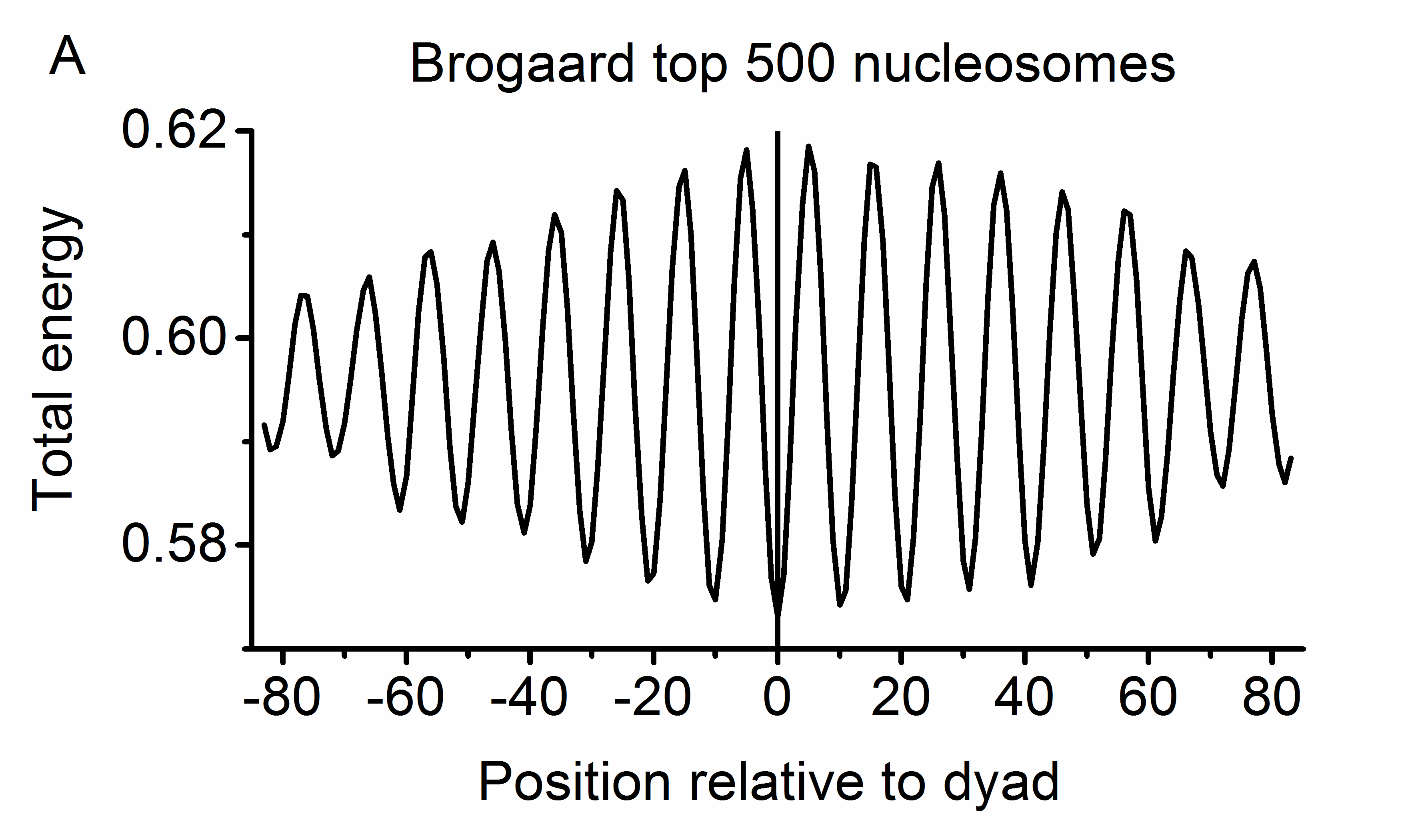
**

**
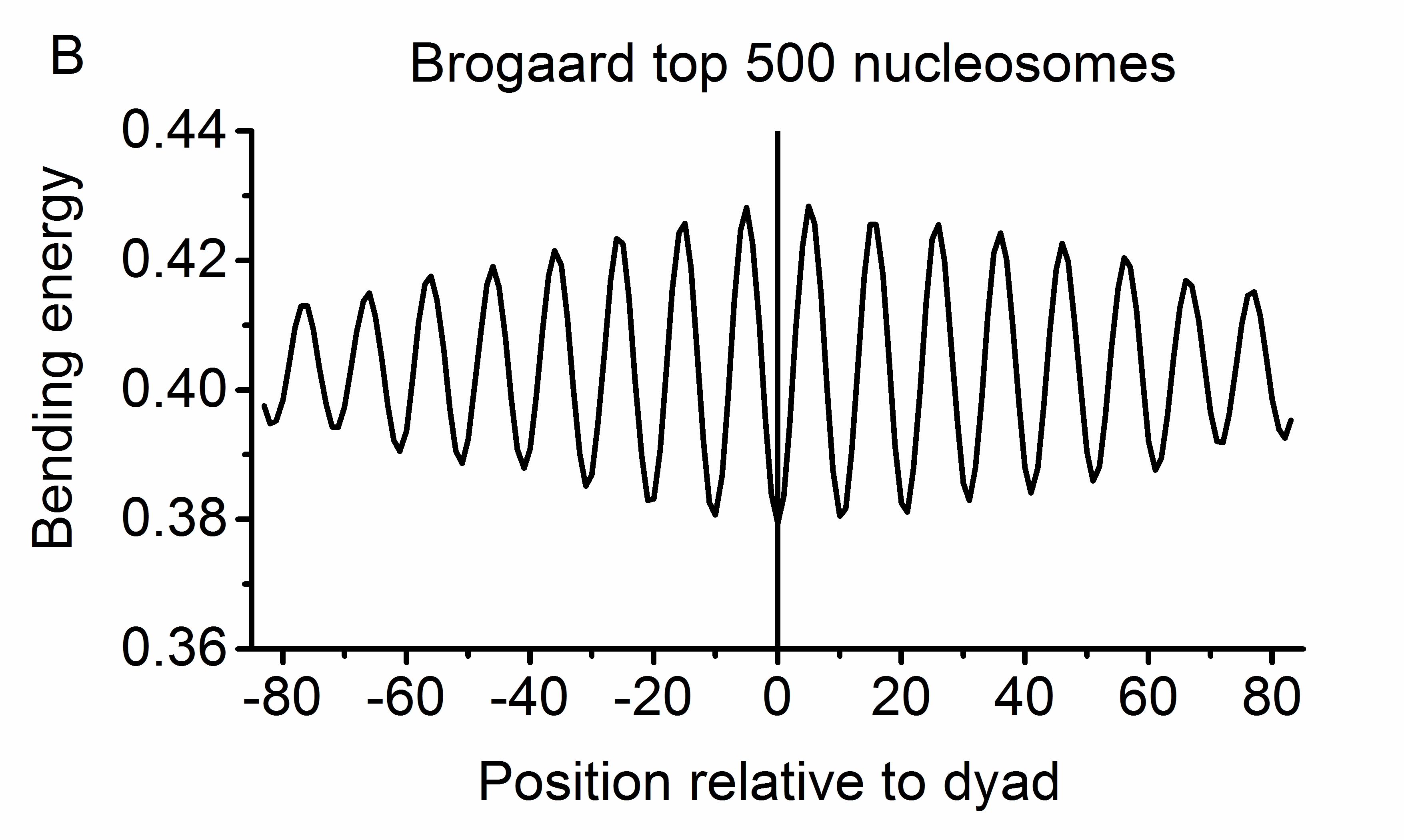
**

**
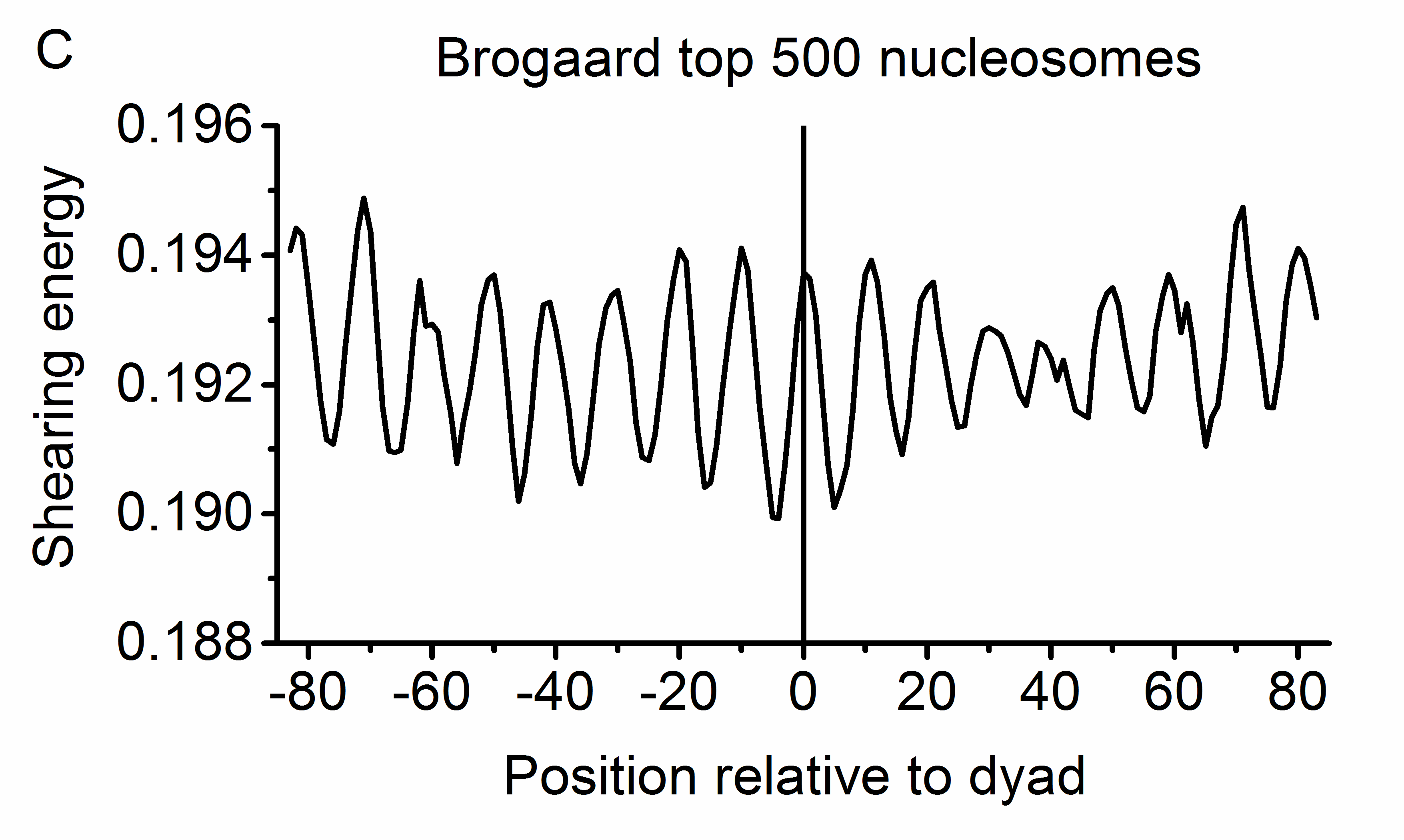
**

Fig S6. Calculated deformation energy profile for top 500 nucleosomal sequences taken from in vivo unique map produced by Brogaard et al.^34^.

A


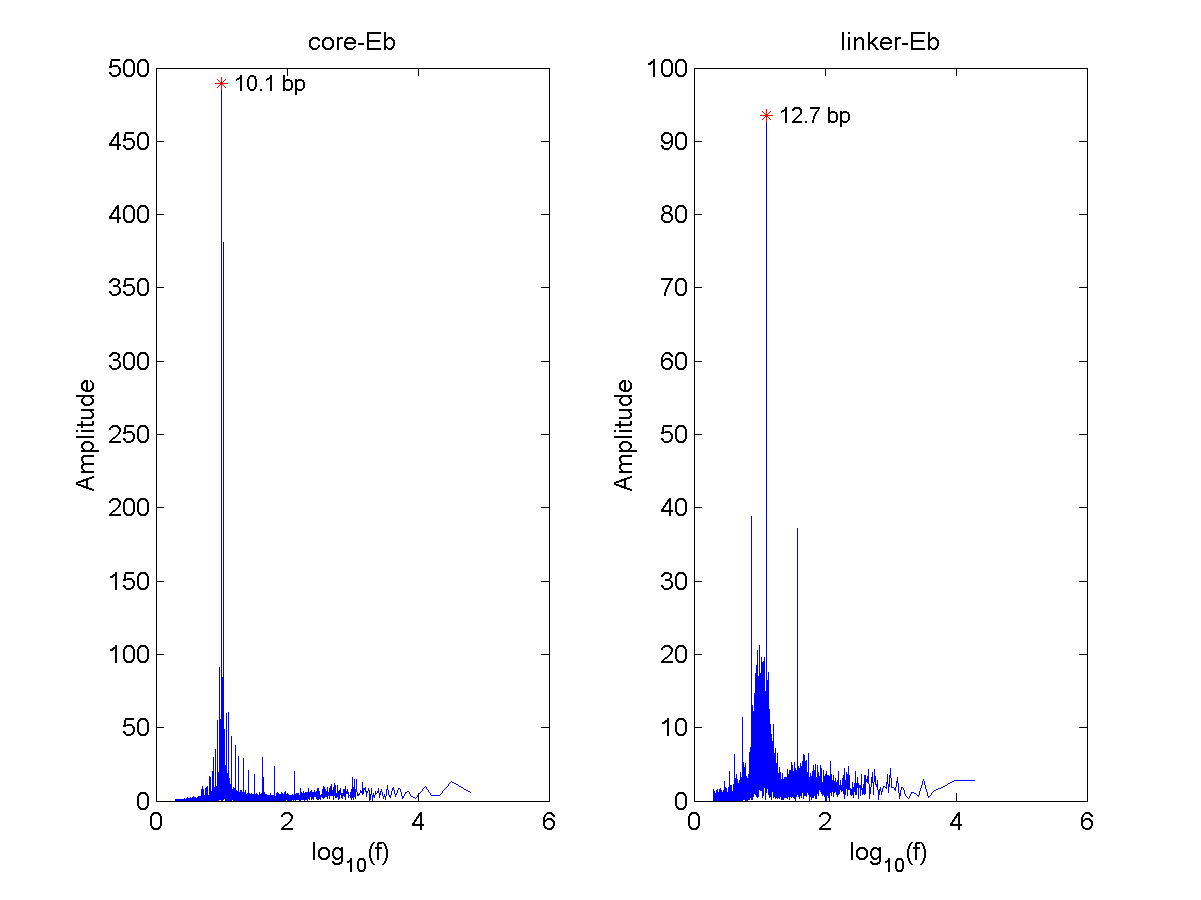


B


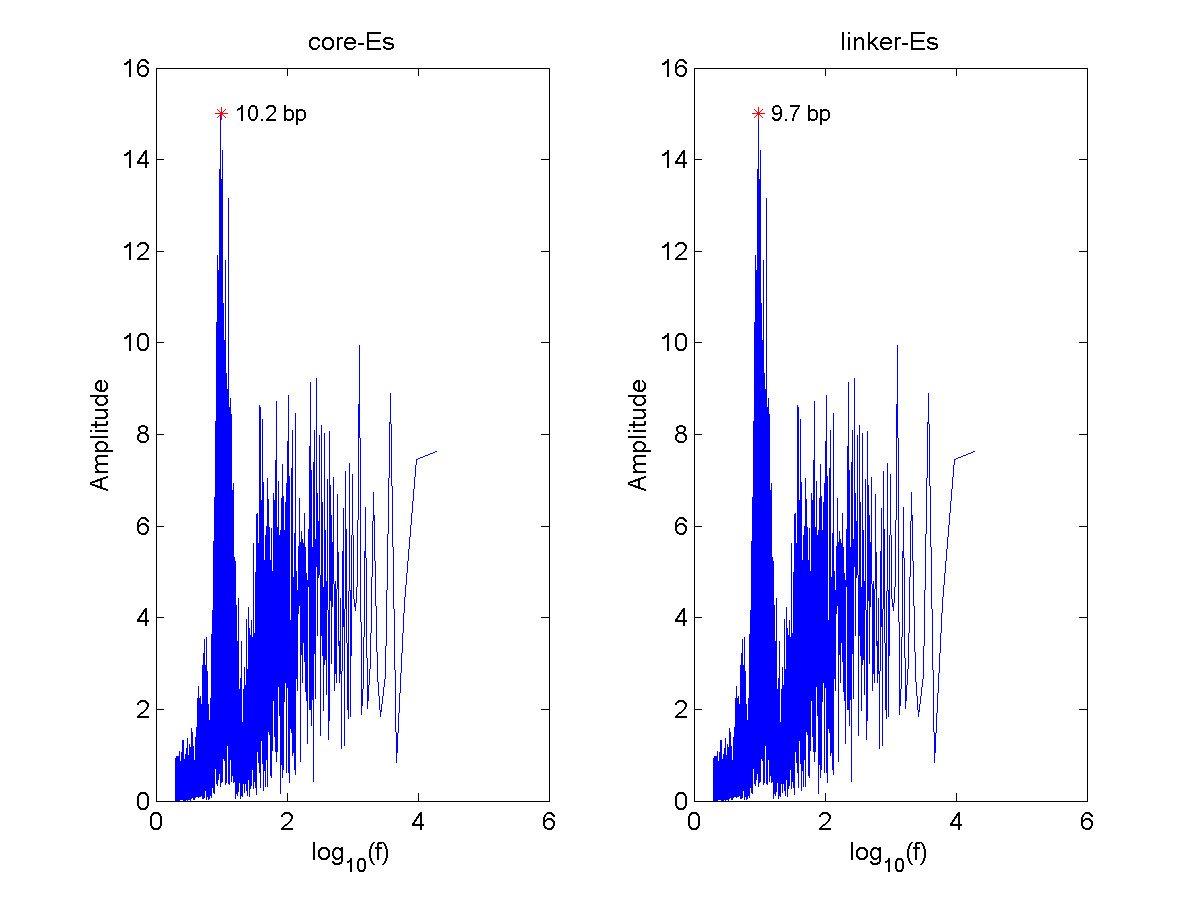


C


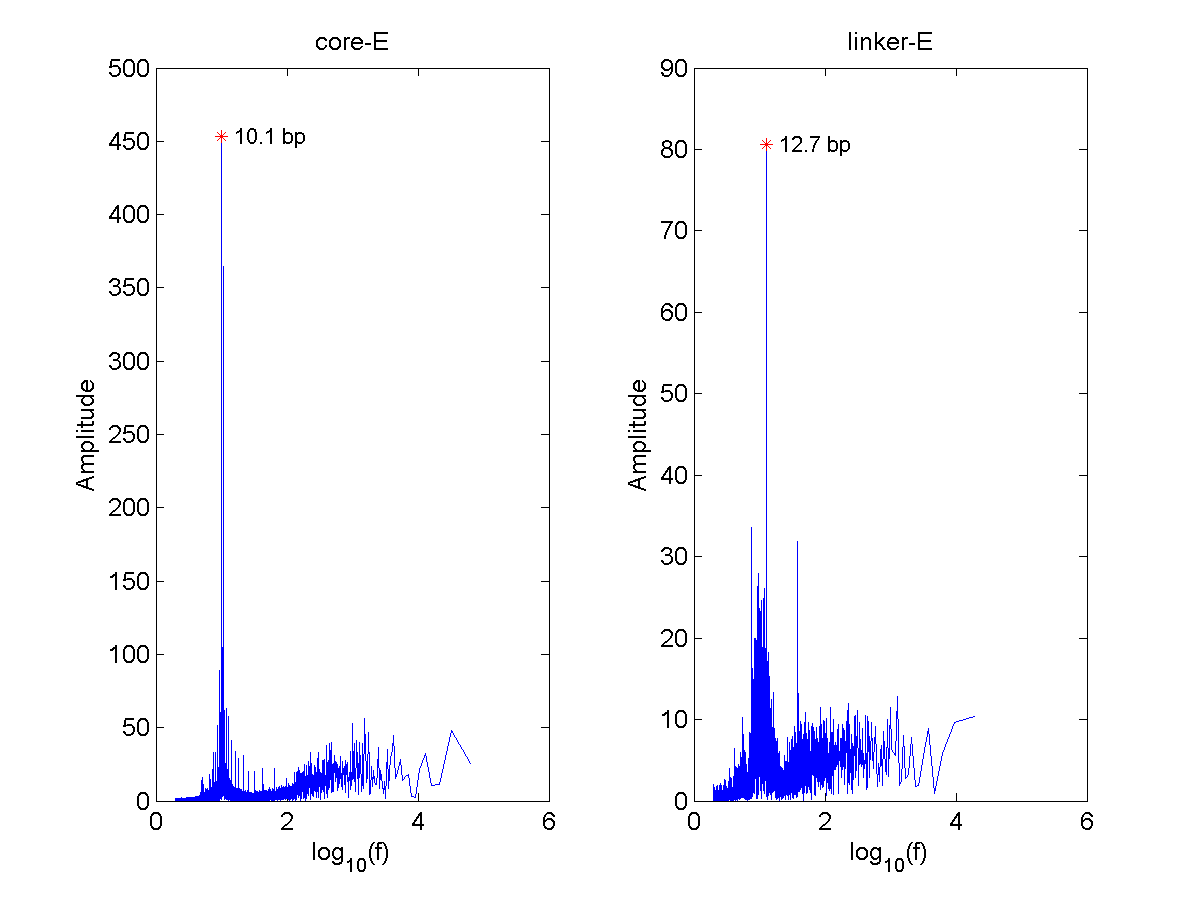


Fig S7. Amplitude of the Fourier transform of deformation energy (A: bending energy, B: shearing energy, C: total deformation energy) vs logarithm of periodicity for top 500 nucleosomal sequences taken from in vivo unique map produced by Brogaard et al^34^. Periodicities correspond to spikes above the baseline in the plots. Each plot corresponds to the whole dataset of deformation energies for analyzed sequences. When Fourier transform was performed for each sequence segment analyzed, the ~10-bp periodicity in energy profile is significantly stronger (*t*-test: *P*=7.3e-166 and *P*=2.3e-150 for bending energy and total deformation energy, respectively) in nucleosome core regions (central 129 bp) than in flanking regions (flanking 19 bp at each end, denoted as linker in the plots). Periodicity of ~10-bp is more pronounced in bending energy than in shearing energy for nucleosome core regions. For a large number of sequence segments, Fourier transform is unable to detect clear ~10-bp periodicity in shearing energy, and accordingly no *t*-test is implemented for shearing energy-related data.


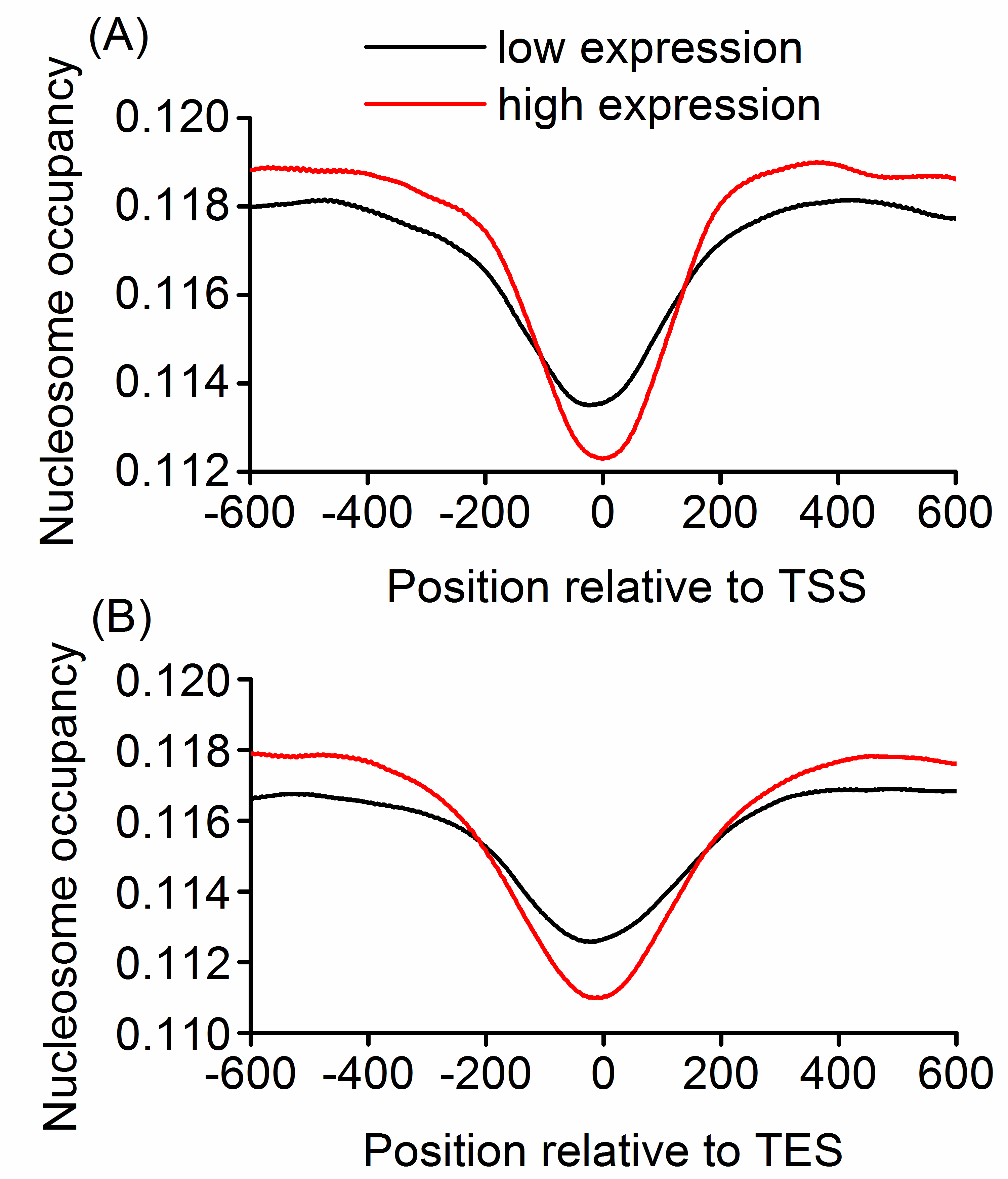


Fig S8. Predicted nucleosome occupancy around (A) transcription start sites (TSS) and (B) transcription end sites (TES). Depletion of nucleosomes was reproduced at both upstream of the TSS and downstream of TES. Model parameters used in the calculation: $\tau=0.001$,$\beta=15$.


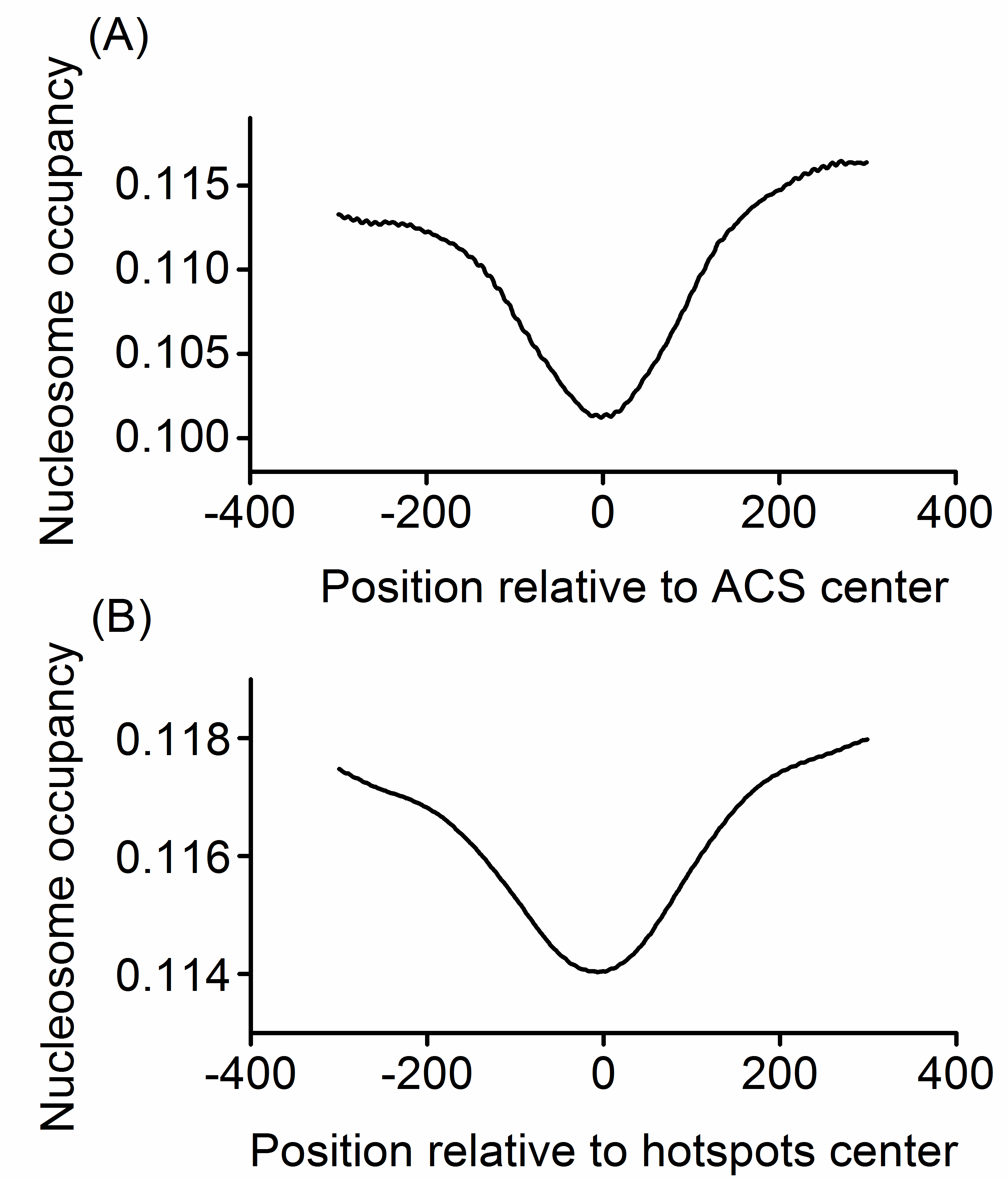


Fig S9. Predicted nucleosome occupancy at (A) consensus sequences (ACS) of replication origins and (B) recombination hotspots. Depletion of nucleosomes was reproduced as observed by others. Model parameters used in the calculation: $\tau=0.001$,$\beta=15$.

Table S1. Dinucleotide-dependent force constants and equilibrium structure parameters obtained in this study. Dinucleotides are counted from single-strands of sequences. The counts of complementary dinucleotides (N) are aggregated in force constants calculation, but not in equilibrium tilts and shifts calculation.

| step | N | *k_ρ_* | *k_τ_* | *k_sl_* | *k_sh_* | *ρ*_0_ | *τ*_0_ | *sl*_0_ | *sh*_0_ | *ω*_0_ |
| --- | --- | --- | --- | --- | --- | --- | --- | --- | --- | --- |
| AA/TT | 3133/3018 | 0.041 | 0.078 | 6.689 | 6.238 | 1.05 | -1.33/1.33 | -0.176 | 0/0 | 35.02 |
| AT | 3298 | 0.054 | 0.098 | 9.611 | 4.657 | 0.61 | 0 | -0.679 | 0 | 30.72 |
| AG/CT | 2524/2390 | 0.042 | 0.058 | 3.472 | 2.801 | 3.60 | -1.58/1.58 | -0.223 | 0.123/-0.123 | 32.29 |
| AC/GT | 2354/2464 | 0.065 | 0.071 | 6.803 | 2.911 | 2.00 | 0.24/-0.24 | -0.593 | 0.252/-0.252 | 31.53 |
| TA | 2255 | 0.031 | 0.065 | 1.853 | 4.107 | 3.50 | 0 | 0.044 | 0 | 36.94 |
| TG/CA | 2833/2829 | 0.035 | 0.056 | 2.004 | 2.882 | 5.60 | 0.10/-0.10 | 0.481 | 0.136/-0.136 | 35.43 |
| TC/GA | 2501/2765 | 0.049 | 0.065 | 4.268 | 3.580 | 2.44 | 1.46/-1.46 | -0.046 | 0.275/-0.275 | 35.67 |
| GG/CC | 2600/2331 | 0.041 | 0.057 | 2.991 | 2.670 | 4.68 | -0.75/0.75 | -0.166 | -0.067/0.067 | 33.54 |
| GC | 2956 | 0.054 | 0.065 | 4.206 | 2.655 | 1.70 | 0 | -0.190 | 0 | 34.06 |
| CG | 2159 | 0.039 | 0.059 | 2.713 | 3.019 | 6.02 | 0 | 0.443 | 0 | 33.67 |

Note: equilibrium tilts and shifts were calculated through three steps. Firstly, average values of tilts and shifts were calculated separately for complementary dinucleotides. Secondly, absolute values of the equilibrium tilts and shifts were averaged over each pair of complementary dinucleotides. Finally, the complementary dinucleotides were assigned the averaged values with their original signs being unchanged. In the first step, all the calculated average values for complementary dinucleotides have different signs except shifts of AA/TT dinucleotides. Therefore, both the AA/TT dinucleotides were assigned zero shifts. These operations aim to reflect the possible conformational anisotropy relative to the two complementary dinucleotides and ensure the consistence of deformation energy calculated on Watson and Crick strand. Note that the modifications to the tilts and shifts have little effect on our results since the bending energy depends more strongly on roll than tilt and the shearing energy depends more strongly on slide than shift.

Table S2. Dinucleotide-dependent force constants and equilibrium structure parameters from Morozov et al.^53^

| step | *k_ρ_* | *k_τ_* | *k_sl_* | *k_sh_* | *ρ*_0_ | *τ*_0_ | *sl*_0_ | *sh*_0_ | *ω*_0_ |
| --- | --- | --- | --- | --- | --- | --- | --- | --- | --- |
| AA/TT | 0.069 | 0.406 | 8.241 | 11.948 | 0.76 | -1.84 | -0.21 | -0.05 | 35.31 |
| AT | 0.124 | 0.641 | 15.942 | 6.670 | -1.39 | 0 | -1.39 | 0 | 31.21 |
| AG/CT | 0.077 | 0.280 | 4.561 | 4.954 | 3.15 | -1.48 | -0.27 | 0.12 | 33.05 |
| AC/GT | 0.085 | 0.302 | 10.089 | 4.912 | 0.91 | -0.64 | -0.54 | 0.21 | 31.52 |
| TA | 0.064 | 0.365 | 4.961 | 8.767 | 5.25 | 0 | 0.03 | 0 | 36.20 |
| TG/CA | 0.059 | 0.393 | 2.772 | 5.740 | 5.95 | -0.05 | 0.18 | -0.16 | 35.02 |
| TC/GA | 0.097 | 0.408 | 6.278 | 10.434 | 3.87 | -1.52 | -0.03 | -0.27 | 34.80 |
| GG/CC | 0.075 | 0.218 | 3.795 | 3.917 | 3.86 | 0.4 | -0.47 | 0.02 | 33.17 |
| GC | 0.057 | 0.256 | 4.030 | 3.473 | 0.67 | 0 | -0.07 | 0 | 34.38 |
| CG | 0.04 | 0.255 | 3.991 | 3.104 | 4.25 | 0 | 0.57 | 0 | 35.30 |

Table S3. Dinucleotide-dependent force constants and equilibrium structure parameters from Olson et al.^68^

| step | *k_ρ_* | *k_τ_* | *k_sl_* | *k_sh_* | *ρ*_0_ | *τ*_0_ | *sl*_0_ | *sh*_0_ | *ω*_0_ |
| --- | --- | --- | --- | --- | --- | --- | --- | --- | --- |
| AA/TT | 0.049 | 0.100 | 6.160 | 3.978 | 0.7 | -1.4 | -0.08 | -0.03 | 35.1 |
| AT | 0.055 | 0.166 | 10.694 | 3.172 | 1.1 | 0 | -0.59 | 0 | 29.3 |
| AG/CT | 0.096 | 0.149 | 7.187 | 3.205 | 4.5 | -1.7 | -0.25 | 0.09 | 31.9 |
| AC/GT | 0.080 | 0.111 | 6.366 | 2.944 | 0.7 | -0.1 | -0.58 | 0.13 | 31.5 |
| TA | 0.029 | 0.148 | 2.350 | 3.860 | 3.3 | 0 | 0.05 | 0 | 37.8 |
| TG/CA | 0.048 | 0.082 | 2.395 | 3.733 | 4.7 | 0.5 | 0.53 | 0.09 | 37.3 |
| TC/GA | 0.046 | 0.087 | 2.780 | 6.542 | 1.9 | -1.5 | 0.09 | -0.28 | 36.3 |
| GG/CC | 0.064 | 0.119 | 3.542 | 2.425 | 3.6 | -0.1 | -0.22 | 0.05 | 32.9 |
| GC | 0.082 | 0.082 | 6.244 | 3.350 | 0.3 | 0 | -0.38 | 0 | 33.6 |
| CG | 0.050 | 0.068 | 3.301 | 1.586 | 5.4 | 0 | 0.41 | 0 | 36.1 |

Table S4. Pearson correlations between nucleosome occupancy predicted by using different sets of parameters and experimentally-determined in vitro nucleosome occupancy^20^ along the yeast chrIII.

|  | Liu | Olson | Morozov | Olson-Liu | Liu-Olson |
| --- | --- | --- | --- | --- | --- |
| Bending | 0.641 | -0.685 | 0.609 | -0.671 | 0.611 |
| Shearing | 0.818 | 0.718 | 0.821 | 0.715 | 0.815 |
| Total | 0.791 | -0.294 | 0.734 | -0.278 | 0.787 |

Note: ‘Liu’, ‘Olson’ and ‘Morozov’ represent dinucleotide physical parameters obtained in this study, from Olson et al.^68^, and Morozov et al.^53^, respectively. ‘Olson-Liu’ means the elastic force constants are taken from Olson et al.^68^ and equilibrium structure parameters are those obtained in this study. ‘Liu-Olson’ means the elastic force constants are those obtained in this study and equilibrium structure parameters are taken from Olson et al.^68^. All the correlations in the table are significant at the level *P*<0.0001. Nucleosome occupancy was predicted by Boltzmann model using shearing energy as input. Grand canonical model achieved similar results (data not shown).

Table S5. Spearman rank correlations of our force constants with other sets of force constants for 10 unique dinucleotides

|  | with Morozov | with Olson |
| --- | --- | --- |
| *k_ρ_* | 0.527 | **0.648** |
| *k_τ_* | 0.600 | 0.255 |
| *k_sl_* | **0.794** | **0.758** |
| *k_sh_* | **0.733** | 0.382 |

Note: significant correlations (*P*<0.05) are indicated in bold.

Table S6. The genomic positions of ACS in yeast used in this study

| chromosome | start | end |
| --- | --- | --- |
| chrI | 159953 | 159967 |
| chrIII | 1050 | 1060 |
| chrIII | 11257 | 11267 |
| chrIII | 14701 | 14711 |
| chrIII | 15186 | 15196 |
| chrIII | 15298 | 15308 |
| chrIII | 30429 | 30439 |
| chrIII | 39579 | 39589 |
| chrIII | 74521 | 74531 |
| chrIII | 108960 | 108970 |
| chrIII | 132044 | 132054 |
| chrIII | 166659 | 166669 |
| chrIII | 292671 | 292681 |
| chrIII | 294821 | 294831 |
| chrIV | 46217 | 46227 |
| chrIV | 462594 | 462604 |
| chrIV | 1159442 | 1159456 |
| chrIV | 1166162 | 1166176 |
| chrVI | 5482 | 5492 |
| chrVI | 32708 | 32718 |
| chrVI | 32960 | 32970 |
| chrVI | 68827 | 68837 |
| chrVI | 118673 | 118683 |
| chrVI | 127864 | 127874 |
| chrVI | 136032 | 136042 |
| chrVI | 167719 | 167729 |
| chrVI | 199404 | 199414 |
| chrVI | 216472 | 216482 |
| chrVI | 256362 | 256372 |
| chrVII | 64444 | 64458 |
| chrVII | 112129 | 112143 |
| chrVII | 163242 | 163256 |
| chrVII | 203978 | 203992 |
| chrVII | 286010 | 286024 |
| chrVII | 388835 | 388849 |
| chrVII | 421271 | 421285 |
| chrVII | 485101 | 485115 |
| chrVII | 568650 | 568664 |
| chrVII | 659990 | 660004 |
| chrVII | 715319 | 715333 |
| chrVII | 778019 | 778033 |
| chrVII | 834655 | 834669 |
| chrVII | 888418 | 888432 |
| chrVII | 977896 | 977910 |
| chrXII | 459079 | 459089 |
| chrXII | 468216 | 468226 |
| chrXVI | 777082 | 777096 |

**Text S1. In vitro nucleosomal sequences taken from Cui et al.^57^**

>601

AAAGCATGATTCTTCACACCGAGTTCATCCCTTATGTGATGGACCCTATACGCGGCCGCCCTGGAGAATCCCGGTGCCGAGGCCGCTCAATTGGTCGTAGCAAGCTCTAGCACCGCTTAAACGCACGTACGCGCTGTCCCCCGCGTTTTAACCGCCAAGGGGATTACTCCCTAGTCTCCAGGCACGTGTCAGATATATACATCCTGTGCATGTATTGAACAGCGACCTTGCC

>603

CGAGACATACACGAATATGGCGTTTTCCTAGTACAAATCACCCCAGCGTGACGCGTAAAATAATCGACACTCTCGGGTGCCCAGTTCGCGCGCCCACCTACCGTGTGAAGTCGTCACTCGGGCTTCTAAGTACGCTTAGGCCACGGTAGAGGGCAATCCAAGGCTAACCACCGTGCATCGATGTTGAAAGAGGCCCTCCGTCCTTATTACTTCAAGTCCCTGGGGTACCGTTTC

>605

TACTGGTTGGTGTGACAGATGCTCTAGATGGCGATACTGACAGGTCAAGGTTCGGACGACGCGGGATATGGGGTGCCTATCGCACATTGAGTGCGAGACCGGTCTAGATACGCTTAAACGACGTTACAACCCTAGCCCCGTCGTTTTAGCCGCCCAAGGGTATTCAAGCTCGACGCTAATCACCTATTGAGCCGGTATCCACCGTCACGACCATATTAATAGGACACGCCG

>5S sea urchin

ATCTTCCAACGAATAACTTCCAGGGATTTATAAGCCGATGACGTCATAACATCCCTGACCCTTTAAATAGCTTAACTTTCATCAAGCAAGAGCCTACGACCATACCATGCTGAATATACCGGTTCTCGTCCGATCACCGAAGTCAAGCAGCATAGGGCTCGGTTAGTACTTGGATGGGAT

>5S somatic

CGGGCTTGTTTTCCTGCCTGGGGGAAAAGACCCTGGCATGGGGAGGAGCTGGGCCCCCCCCAGAAGGCAGCACAAGGGGAGGAAAAGTCAGCCTTGTGCTCGCCTACGGCCATACCACCCTGAAAGTGCCCGATATCGTCTGATCTCGGAAGCCAAGCAGGGTCGGGCCTGGTTAGTACTTGGATGGGAGACCGCCTGGGAATACCAGGTGTCGTAGGCTTTTGCACTTTTGCCATTCTGAGT

>5S oocyte

GGAATACCAGGTGTCGTAGGCTTTTAGACTTTTGCCAGGTCAAAGTTTTGCAGGGTTTTTCGTCAAAGTCTTCATAGAAGCGTCAAAAGTCTTCACTCCGATGCCTACGGCCACACCCCCCTGAAAGTGCCCGATCTCGTCTGATCTCGGAAGCGATGCAAGGTCGGGCCTGGTTAGTACCTGGATGGGAGACCGCCTGGGAATACCAGGTGTCGTAGGCTTTTAGACTTTTGCCAGGTCAAAG

>Chicken$\beta$-globin

CTGGTGTGCTGGGAGGAAGGACCCAACAGACCCAAGCTGTGGTCTCCTGCCTCACAGCAATGCAGAGTGCTGTGGTTTGGAATGTGTGAGGGGCACCCAGCCTGGCGCGCGCTGTGCTCACAGCACTGGGGTGAGCACAGGGTGCCATGCCCACACCGTGCATGGGGATGTATGGCGCACTCCGGTATAGAGCTGCAGAGCTGGGAATCGGGGGGGGGGGGGGGGCGGGTGGTGGTGTGGCACTGGATCTGGGCACCTTACTCCTGAGCCCCACCCTGATGCCGCGTTCCCTCCCCGGGGTTCCAAGGCCGGGGCCCTCCGGAAGATGCCACCCAATTCCGGGGTGCCCGGGAAGAGGAGGGGCCCGGGCGGAGCGATAAAAGTGGGGACACAGACGGCCGCTCACCAGCGTGCTATCCCCACGGGAGCAAGAGCCCAGACCTCCTCCGTACCGACAGCCACACGCTACCCTCCAACCGCCGCCATGGTGCACTGGACTGCTGAGGAGAAGCAGCTCATCACCG

>MMTV

ATCAATAGCCTTTATTGGCCCAACCTTGCGGTTCCCAGGGCTTAAGTAAGTTTTTGGTTACAAACTGTTCTTAAAACGAGGATGTGAGACAAGTGGTTTCCTGACTTGGTTTGGTATCAAAGGTTCTGATCTGAGCTCTGAGTGTTCTATTTTCCTATGTTCTTTTGGAATTTATCCAAATCTTATGTAAATGCTTATGTAAACCAAGATATAAAAGAGTGCTGATTTTTTGAGTAAACTTGCAACAGTCCTAACATTCACCTCTTGTGTGTTTGTGTCTGTTCGCCATCCCGTCTCCGCTCGTCACTTATCCTTCACTTTCCAGAGGGTCCCCCCGCAGACCCCGGCGACCCTCAGGTCGGCCGACTGCGGCACAGTTTTTTGCTCCTTTTTCTAGATGTAATTTTTAAAGCTTATTTTTTAACTTTCACATGTGAT

>pGUB

GATCCTCTAGACGGAGGACAGTCCTCCGGTTACCTTCGAACCACGTGGCCGTCTAGATGCTGACTCATTGTCGACACGCGTAGATCTGCTAGCATCGATCCATGGACTAGTCTCGAGTTTAAAGATATCCAGCTGCCCGGGAGGCCTTCGCGAAATATTGGTACCCCATGGAATCGAGGGATC

>Fragment_67

CACAGGACCCAGCTATGACCATGATTACGAATTCCGGCAAAAACCGGCAAAAACGCGGAAAAAGCCGCAAAAACTCGAGGGTACCCGGGGATCCTCTAGAGTCTAGATCTGTCGACTGCAGTCGACCTGCAGGCCAAAAACGCGCAAAAACCCGCAAAAAGGCCGAAAAATGATCACAAGCTTGGCACTGGCCGTCGTTTTACAACGTCGTGACTGGGACCCCCC

**Supplementary Methods:**

**1. Grand canonical model for nucleosome formation**

Nucleosome formation (assembly of histone octamers along a DNA sequence) is modeled as a particle absorbing process along the DNA sequence. There are a large number of possible states that nucleosomes are assembled along a long DNA sequence. Partition function for this grand-canonical ensemble describes all possible configurations of nucleosome assembly along a sequence. For a system that consists of nucleosomes of *M* bp distributed along a DNA segment of *N* bp, the grand-canonical partition function is given by

$Z=\sum_{conf} e^{-\beta[E(conf)-\mu n(conf)]}$ (1)

where $conf$ denotes an arbitrary configuration of DNA-bound non-overlapping nucleosomes, $\mu$ is the chemical potential, and$E(conf)$and $n(conf)$ are the total energy of an arbitrary configuration of non-overlapping particles and the number of particles in the current configuration.

Partition function Z can be efficiently evaluated by recursively computing a set of partial partition functions in the forward direction,

$Z_{j}^{f}=\left\{\begin{matrix} 1\text{ , }j=0,1,...,M-1\text{ } \\ Z_{j-1}^{f}+Z_{j-M}^{f}e^{-\beta(E_{j-M+1}-\mu)}\text{ , }j=M,M+1,...,N \end{matrix}\right.$ (2)

Similarly, partial partition functions in the reverse direction can be computed by

$Z_{j}^{r}=\left\{\begin{matrix} 1\text{ , }j=N-M+2,N-M+3,...,N+1\text{ } \\ Z_{j+1}^{r}+Z_{j+M}^{r}e^{-\beta(E_{j}-\mu)}\text{ , }j=N-M+1,N-M,...,1 \end{matrix}\right.$ (3)

Note that $Z_{N}^{f}=Z_{1}^{r}=Z$. The probability of a nucleosome to start at position *j* is computed by

$P_{j}=\frac{Z_{j-1}^{f}e^{-\beta(E_{j}-\mu)}Z_{j+M}^{r}}{Z}$ (4)

In this study, $M=147$ (nucleosome size) and$E_{j}$is the deformation energy of the underlying DNA of the nucleosome which occupies positions *j* through *j+M-*1.

It is apparent that $P_{j}$can also denote dyad probability defined as the probability of a nucleosome to center at position *j+*73.

The nucleosome occupancy at base-pair *j* is defined as the probability that base-pair *j* is covered by any nucleosome:

$O_{j}=\sum_{i=j-(L-1)}^{j} P_{i}$ (5)

where $P_{i}=0$ when$i<1$and $i>N-M+1$.

There are two parameters in the model, inverse temperature$\beta$ and chemical potential$\mu$. The chemical potential reflects nucleosome concentration, thereby being related to the average level of nucleosome occupancy genome-wide. Inverse temperature determines the variance of genome-wide nucleosome occupancy. The procedure of the determination of the parameters is described below.

**2. Parameter adjustment**

The term $e^{-\beta(E_{j}-\mu)}$can be re-written as $e^{-\beta(E_{j}-\mu)}\text{=}e^{\beta(\mu_{1}\text{+}\mu_{2}-E_{j})}=e^{\beta\mu_{1}}e^{\beta(\mu_{2}-E_{j})}$, where $\mu=\mu_{1}+\mu_{2}$. Let $\tau=e^{\beta\mu_{1}}$, $\mu_{2}=\overline{E}_{j}$, where $\overline{E}_{j}$denotes the mean of deformation energy $E_{j}$, then we have $e^{-\beta(E_{j}-\mu)}=\tau e^{\beta(\overline{E}_{j}-E_{j})}$. For a given inverse temperature, $\tau$is a modulating factor that determines the chemical potential $\mu$.

There are several concerns in the parameter adjustment. Firstly, $\beta$cannot be too large, otherwise, obtained dyad probability has a large variance, resulting in the cluster of dyad probability values at two extremes. This would cause the over-estimation or even mis-estimation of the difference in relative strength of both dyad probability and nucleosome occupancy for different nucleotide positions. Secondly, while it remains theoretically plausible that$\tau$factor can be modulated to simulate average level of experimental nucleosome coverage genome-wide, it is not practically useful as it usually cannot generate an ideal global correlation of prediction with experimental occupancy. We suggest that the relative strengths of both dyad probability and nucleosome occupancy are more important than their absolute values. For example, if we adjust the dyad probability by tuning inverse temperature which acts as a modulator of variance of deformation energy to obtain stable nucleosomes (defined as those with dyad probability larger than 0.5 in Morozov et al.^53^) covering ~17% of the yeast genome, we cannot obtain successful prediction of nucleosome occupancy. It is possible that the improper setting of the parameters (namely,$\mu$was set to the mean of their deformation energies to obtain genome-wide nucleosome coverage of 0.797, and the variance of deformation energies was rescaled to 45 to simulate the amount of stable nucleosomes in yeast) is the cause of the poor prediction of nucleosome occupancy by Morozov et al.^53^.

Accoriding to the concerns described above, we obtained several sets of parameters: the first set is for predicting in vitro nucleosome dyads ($\tau=0.35$,$\beta=35$) using bending energy. In this set,$\tau$factor was determined to obtain a nucleosome occupancy maximum close to 1, and$\beta$was determined to make the minimum dyad probability close to zero. The other sets are for predicting genome-wide nucleosome occupancy based on bending energy ($\tau=0.001$,$\beta=0.1$), shearing energy ($\tau=0.001$,$\beta=15$) and total energy ($\tau=0.001$,$\beta=1$), respectively. In these sets, the parameters were determined to obtain nucleosome occupancy data that best fit the experimentally-determined nucleosome occupancy data of Kaplan et al.^20^. In the modeling of data of Zhang et al.^27^, we used different parameters ($\tau=0.01$,$\beta=15$) to obtain best results based on shearing energy. We also predicted nucleosome occupancy in *Caenorhabditis elegans* and *Drosophila melanogaster* using shearing energy ($\tau=0.001$,$\beta=15$) to test the applicability of our model.

Note that the calculated nucleosome occupancy values at both ends (at least 147 bp region at each end) of a sequence cannot reflect a real situation due to boundary effect, and thus cannot be used in correlation analysis between prediction and experimental data. To ensure the elimination of boundary effect, data corresponding to both 1000-bp ends of a chromosome were not used in our correlation analysis.

**3. A simple model to predict nucleosome occupancy based on Boltzmann distribution**

Apart from method described above, a simple way to assess the nucleosome-forming ability of a DNA segment without considering steric exclusion is Boltzmann distribution law. As deformation energy is negatively correlated with nucleosome formation probability, the probability that a nucleosome occupies nucleotide positions *j* through *j+M-*1 in a DNA segment of *N* bp is defined according to Boltzmann distribution:

$S_{j+(M-1)/2}=\frac{e^{-\beta E_{j}}}{Z}$ , $j=1,2,...,N-M+1$ (6)

where$M=147$ (nucleosome size), Z is partition function, and$E_{j}$is the deformation energy of the underlying DNA of the nucleosome which occupies positions *j* through *j+M-*1.

Nucleosome occupancy at the *j*th base-pair site is given simply by smoothing the nucleosome formation probability with *l*-bp window (*l*=147),

$O_{j}=\sum_{i=j-(l-1)/2}^{j+(l-1)/2} S_{i}/l$ (7)

Note that this equation leads to averaging effect of deformation energy.

Normalized nucleosome occupancy at every base-pair is calculated by the log-ratio between the corresponding absolute nucleosome occupancy $O_{j}$ and the average nucleosome occupancy$\overline{O}_{j}$ per base-pair across the genome as

$O_{j}^{nor}=\log\frac{O_{j}}{\overline{O}_{j}}\text{=}\log\frac{O_{j}}{\overline{O}_{j}}$ (8)

In the final equation of normalized nucleosome occupancy, partition function does not occur and, for simplicity, we assume$\beta=1$ in calculation.

**4. Fourier transform**

Fourier Transform is a popular method for detecting periodicity in numerical sequences. To accelerate calculation, we used Fast Fourier Transform algorithm to compute power spectrum. For N-sized numerical data,$E(m)$, (eg. deformation energy), its Fourier transform$F(k)$is given by

$F(k)=\sum_{m=0}^{N-1} E(m)e^{-2\pi mik/N}$, $k=0,1,\cdots,N-1$ (9)

Where N is a positive even number. Periodicity is denoted as$f=N/k$. Practically, it is enough to analyze the Fourier transform with *k* in the range$\left[0,N/2\right]$, as the amplitude of the Fourier transform,$\left|F(k)\right|$, is symmetric with respect to$k=N/2$.

**5. Reads data filtering**

Reads data of Zhang et al.^27^ were filtered to eliminate possible sequencing artifacts, similarly as described in Locke et al.^69^. Any stretch of ≥1000 bp without mapped reads was excluded. Then the regions with extremely high read coverage values were excluded as follows: the genome-wide mean of reads per base-pair and for each base-pair, running average of reads in a window of 75 bp were calculated. If the running average is more than three times the genome-wide mean, corresponding region was excluded from analysis. Nucleosome occupancy for each base-pair was defined simply as the filtered read coverage.

**6. Classification of nucleosome-forming regions and nucleosome-inhibiting regions**

To assess the ability of our model in discriminating nucleosome-forming regions and nucleosome-inhibiting regions, we made some predictions on the sequences described below.

For *Saccharomyces cerevisiae*, nucleosome-enriched regions and nucleosome-depleted regions were defined as Kaplan et al. did^20^. According to the in vitro (or in vivo) nucleosome map^20^, nucleosome-enriched regions were defined as maximal consecutive regions longer than 50 bp for which nucleosone occupancy at every site is above a threshold of 0.75, and nucleosome-depleted regions as maximal consecutive regions longer than 50 bp for which nucleosome occupancy at every site is below a threshold of -0.75. Following this criterion, we obtained 17,303 (or 16,683) nucleosome-enriched regions and 12,145 (or 11,055) nucleosome-depleted regions based on in vitro (or in vivo) map. Because of the abnormally high disagreement between the experimental nucleosome map and the predicted nucleosome occupancy for chrX in yeast as discussed in this study (Table 2), the data on chrX was not considered in the definition of nucleosome-enriched and nucleosome-depleted regions.

In addition, we also tested our model using nucleosome-forming sequences and nucleosome-inhibiting sequences in *Saccharomyces cerevisiae* and *Drosophila melanogaster*, which were defined in a previous study^59^. To make a comparison, we predicted the nucleosome-forming sequences and nucleosome-inhibiting sequences exactly in the same way as described in Liu et al.^59^: 100 sequences were randomly selected from each class (nucleosome-forming or nucleosome-inhibiting) of sequences, and calculated nucleosome occupancies at midpoints of the selected sequences were used to predict their class by using an adjustable threshold value of the nucleosome occupancies. This procedure was repeated 100 times and the average performance was reported by the mean of the AUC **(A**rea **U**nder the ROC **C**urve) values of the 100 tests. AUC equals 1 for perfect prediction and 0.5 for random guessing.
